# Influence of Hydrothermal and Chemical Modifications of Potato Starch on Its Performance as a Carrier Matrix for Selected Polyphenolic Compound Derived from Chokeberry (*Aronia melanocarpa*) Fruit

**DOI:** 10.3390/molecules31173110

**Published:** 2026-09-04

**Authors:** Justyna Kobryń, Eliza Moczurad, Małgorzata Kapelko-Żeberska, Tomasz Zięba, Witold Musiał

**Affiliations:** 1 Department of Physical Chemistry and Biophysics, Wrocław Medical University, Borowska 211A, 50-556 Wrocław, Poland; justyna.kobryn@umw.edu.pl (J.K.); elizaannamoczurad@interia.pl (E.M.); 2 Department of Food Storage and Technology, Faculty of Biotechnology and Food Science, Wrocław University of Environmental and Life Sciences, Chełmońskiego 37, 51-630 Wrocław, Poland; malgorzata.kapelko@upwr.edu.pl (M.K.-Ż.); tomasz.zieba@upwr.edu.pl (T.Z.); 3 Institute of Sport, Tourism and Nutrition, Faculty of Biological Sciences, University of Zielona Góra, Licealna 9, 65-417 Zielona Góra, Poland

**Keywords:** modified potato starch, aronia extract, carrier

## Abstract

Starch, a natural source of energy in the form of glucose chains, is widely utilized in various industrial and scientific fields. In its native state, starch is thermally unstable and undergoes gelatinization. Physicochemical modifications of starch aim to increase its thermal and structural stability while simultaneously enhancing its reactivity by introducing new functional groups. The primary objective of this study was to develop thermally stable and economically viable starch-based drug carriers capable of the controlled release of a negatively charged component sourced from aronia extract. Potato starch underwent a series of chemical modifications, specifically quaternary amine etherification, citric acid esterification, and/or hydrothermal modification. The characterization involved determining several parameters: the degree of amino substitution groups; starch particle size using a laser particle size analyzer; viscosity and pH; gelation temperature and heat capacity measured by scanning calorimetry (DSC); mass degradation analyzed via thermogravimetric analysis (TG); crystallinity determined by X-ray diffraction (XRD); potential intermolecular interactions studied by Fourier-Transform Infrared Spectroscopy with Attenuated Total Reflectance (FTIR-ATR); and the rate of chlorogenic acid release from aronia extract tablets quantified by spectrophotometry. The highest cationization results were achieved using citrate starches, reaching up to 86%. The combined application of citric acid esterification and cationization, coupled with an annealing process, resulted in increased viscosity, amorphousness, and enzyme resistance of the starch. Citric acid esterification significantly improved the thermal stability of the starch. Furthermore, FTIR studies revealed the formation of electrostatic interactions between the functional groups of the starch and the components of aronia extract. The amount of chlorogenic acid released showed significant variation (70–100%) depending on the type of starch modification. Collectively, these studies confirmed that both hydrothermal and chemical modifications influence the thermal and structural stability of the starch. Utilizing all combination modification strategies ensured the production of highly promising carriers for active substances.

## 1. Introduction

**Figure 1:**
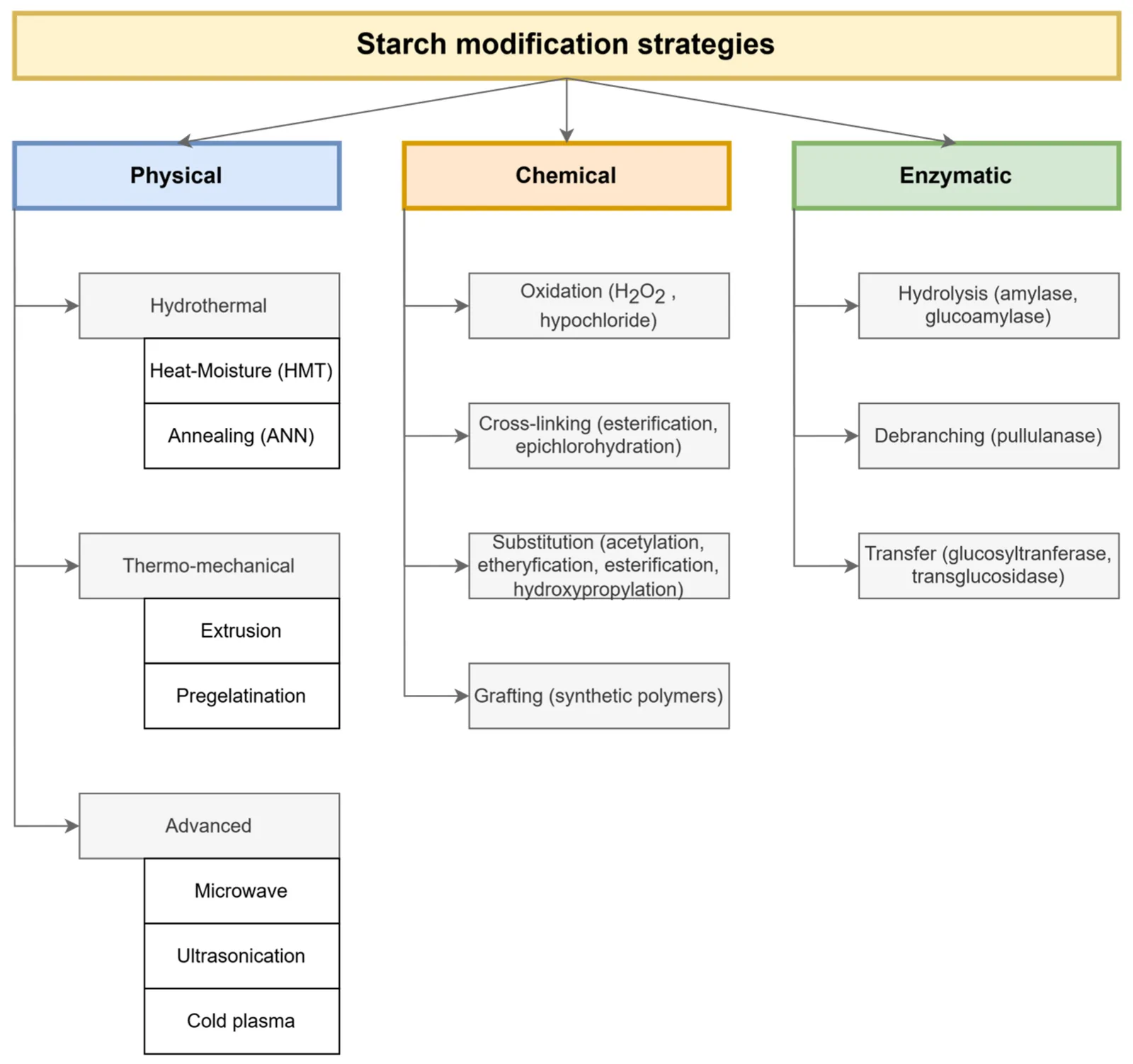
The different starch modification strategies [2,21,22].

The search for natural, inexpensive materials derived from agro-food waste plant parts has driven scientific interest in the properties of starch [1]. Starch obtained from rice, cereals, maize, potatoes, and cassava is widely utilized on an industrial scale. However, native starch possesses inherent limitations, including poor solubility in water, low thermal stability, and high hydrophilicity [2,3,4]. Fortunately, as a glucose polymer, native starch can be relatively easily modified to improve its physicochemical characteristics [5]. Starch solubility can be enhanced through alkaline or thermal modification [6,7] (see Figure 1). For instance, He et al. [8] showed that pre-treating starch with sodium hydroxide facilitates the penetration of water molecules via the presence of the alkaline starch anion. This process increases the spatial separation between starch molecular chains and influences overall aggregation. Specifically, it weakens interactions between crystalline and amorphous regions, allowing the starch anion to bind with sodium ions, resulting in enhanced dissolution and hydration. Similarly, Li et al. [9] investigated using thiourea to prevent spontaneous aggregation of maize starch, while Zhuang et al. [10] used trivalent iron and aluminum cations to reduce the crystalline area of potato starch, thereby boosting its solubility. Chemically modified starches exhibit changes in swelling capacity, gelatinization, and viscosity; for example, Hazarika and Sit [11] showed that hydroxypropylation increased the swelling and solubility of taro starch but decreased its viscosity. Cross-linked starches, conversely, often show reduced swelling and solubility alongside improved adhesive properties. Furthermore, Shen et al. [12] analyzed how cross-linking reactions and hydroxypropylation affect rice starch containing varying amylose amounts, demonstrating that gelatinization capacity, viscosity, and structure are dependent on the initial amylose content. These modifications include starch etherification—the replacement of hydroxyl groups in glucose units with hydroxyalkyl or other groups, forming an ether bond [13]—and cross-linking, which involves reacting the active hydroxyl group of starch with a cross-linking agent (such as acid halides or carboxylic acids). This results in ether or ester bonds formed both between individual and adjacent chains [14,15]. A different category involves physical modifications based on thermal or hydrothermal processes. The heating and subsequent cooling of starch leads to retrogradation. Moreover, the straightening and organization of mainly spiral amylose chains into compact micelles is responsible for forming resistant starch. Thermal treatment affects the physicochemical and digestive properties of starch primarily by altering its structure and the interactions with non-starch components [13,16]. Commonly used processes include heat-moisture treatment (HMT) at temperatures between 90–120 °C (with 10–30% moisture content) and annealing (ANN), which involves heating between the gelatinization temperature and glass transition temperature (Tg) [17,18,19]. HMT technology allows control of molecular mobility by limiting water amount [20], while the ANN process increases starch chain molecule mobility, leading to the reorganization and stabilization of crystalline regions within starch granules and improving structural integrity [18].

Aronia extract has garnered significant scientific attention due to its rich composition of bioactive compounds and potential biological effects. Its most characteristic constituents are polyphenols—including anthocyanins, proanthocyanidins, phenolic acids, and flavonols—which are primarily responsible for the high antioxidant capacity of the extract. These compounds not only scavenge free radicals but also modulate inflammatory pathways, cellular signaling, redox balance, and exhibit antibacterial properties [23,24]. The chemical profile of aronia is dominated by anthocyanins (mainly cyanidin glycosides), which contribute to its intense dark color and are highly recognized for strong reducing capabilities. Additionally, chlorogenic and neochlorogenic acids represent important phenolic acids, complemented by quercetin derivatives and polymeric procyanidins that further enhance its functional potential; the synergy among these constituents may underlie aronia’s broad spectrum of biological activities. Beyond polyphenols, aronia can contain vitamins, minerals, organic acids, and sugars depending on extraction methods. However, its scientific relevance remains closely linked to the abundance and diversity of phenolic compounds, which have been associated with cardioprotective, antidiabetic, anti-inflammatory, and chemopreventive properties in experimental studies [25,26,27]. Consequently, aronia extract is increasingly investigated as a promising natural source for health-promoting agents and serves as a valuable material for developing functional foods, nutraceuticals, and phytopharmaceutical formulations.

Therefore, the aim of this study was to develop cost-effective and thermally stable modified potato starches that could function as API carriers, with specific emphasis on their capability to encapsulate and transport chlorogenic acid from *Aronia melanocarpa extract*—a negatively charged, biologically active substance.

## 2. Results and Discussion

### 2.1. Physicochemical Properties of Tested Starches

The prepared starches were categorized into two primary groups based on their base material. Group I comprised non-cationic native starch (NS0) and non-cationic annealed starch (A0), along with cationic counterparts derived from both NS0 and A0 through cationization conducted over a time period ranging from 5 to 24 h. The cationic starches contained amino groups, and the annealing process involved exposing the starch to a temperature of 58 °C for 24 h. Similarly, Group II consisted of non-cationic citrate starch (C0) and non-cationic annealed citrate starch (CA0), supplemented by their respective cationic forms subjected to the same cationization process (5–24 h). Citrate starches were produced through chemical modification with citric acid. Within each main group, two distinct subgroups were established: Subgroup A represented the unannealed starches, while Subgroup B comprised the annealed formulations (see Section 3.2). The analysis of the modified potato starch groups revealed considerable diversity in physicochemical properties, particularly when comparing the different starch classes. Specifically, Group I demonstrated that annealing significantly increased amine substitution efficiency compared to its non-annealed counterpart, as evidenced by the increase in the amination reaction yield (ARE) in Group IIB compared with Group IIA (Table 1). In contrast, Group II showed C10 as the most substituted formulation; moreover, the presence of CIS carboxyl groups during annealing had a mitigating effect on overall starch cationization (Table 2). Furthermore, the hydrodynamic diameter D[4,3] values for Group II were approximately twice those of Group I, suggesting a strong stabilizing interaction mediated by the CIS carboxyl groups in an aqueous medium. Correspondingly, the viscosity values in Group II averaged four times higher than those in Group I, and this increase was observed to be more pronounced as the degree of starch modification increased.

#### 2.1.1. Viscosity Correlations

**Figure 2:**
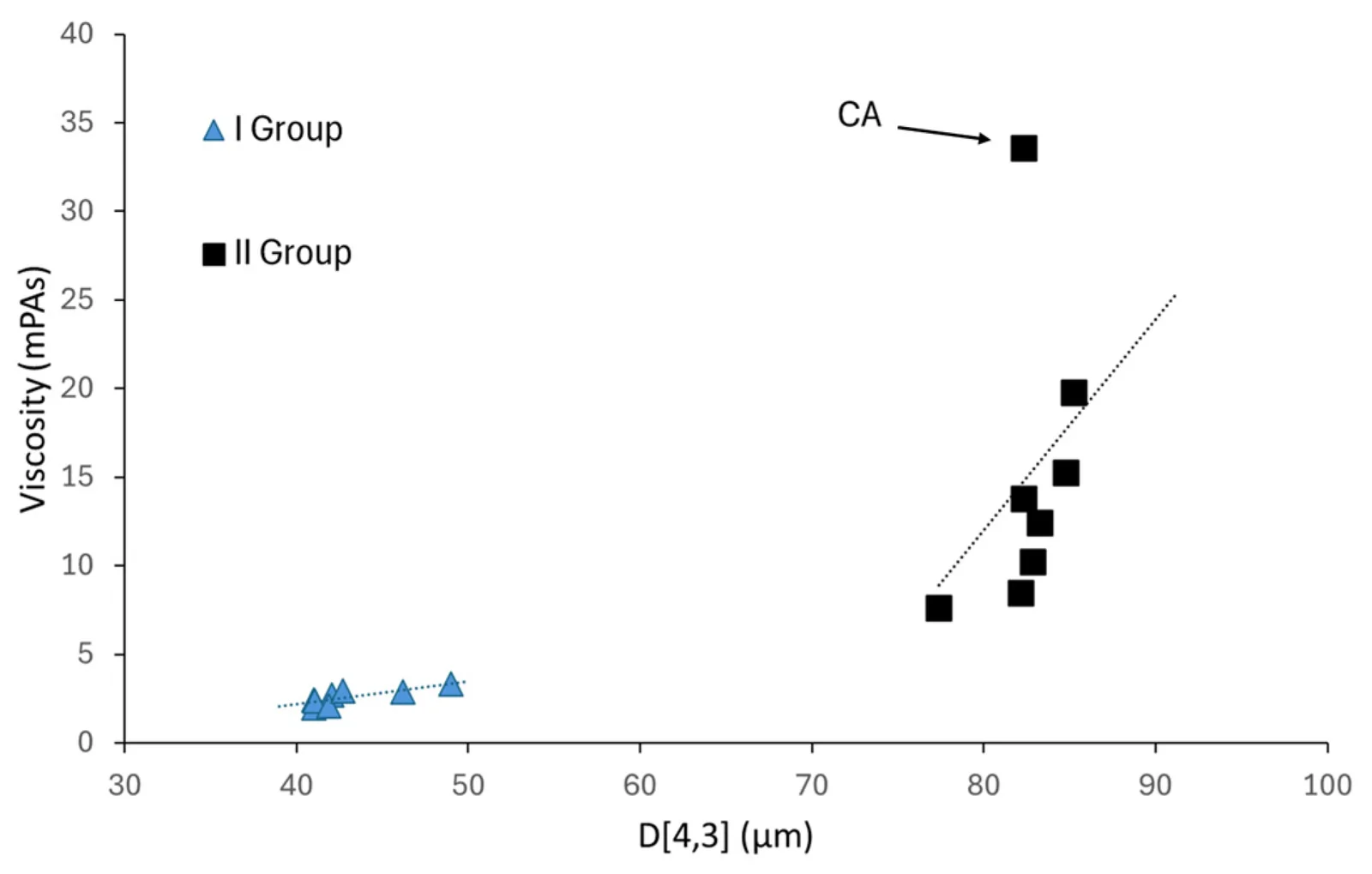
Correlation between viscosity and particle size of the Group I and II of the studied starches at 25 °C.

A correlation was observed between the D[4,3] values and the viscosity of Group I starch. However, an exception was noted in CA5 within Group II (Figure 2). Similarly, Gryszkin et al. [28] reported a relationship between particle size and the viscosity of a native potato starch solution across temperatures ranging from 30 to 94 °C; specifically, increasing particle size corresponded to increased viscosity. This linear relationship was particularly evident for starch heated at 30 °C. In our study, this relationship was nearly linear for Group I, and viscosity increased as particle size increased. In the case of Group II, this relationship was no longer linear, and no clear linear relationship between particle size and viscosity was apparent, particularly for CA5 starch. Given the viscosity observed, CA5 particles should have significantly larger hydrodynamic diameters caused by interactions between carboxyl groups and the aqueous environment, as mentioned. Since this was not the case, it indicates the influence of other factors. Modification of the starch using citric acid at 100 °C resulted in the formation of cross-links between the carboxyl groups of the acid and the hydroxyl groups of the starch. These bonds strengthened the structure of the grain from within. Water penetrating from the outside caused the starch to swell without causing it to rupture [29]. In addition, the annealing process caused the chains to rearrange and become more tightly packed in the amorphous regions [30]. Following a 5-h reaction of annealed citrate starch with an amine, almost 65 percent of the starch was aminated; this yield decreased as the cationization time increased. At the same time, the pH values of the respective starches increased. This suggests a greater affinity of the amine groups for hydroxyl groups than for carboxyl groups. After ten hours of the process, some of the amine groups interacted with the carboxyl groups, blocking them and causing an increase in pH. After twenty-four hours, an equilibrium may have been established between the electrostatic interactions. In the case of annealed citrate starch (CA0), high viscosity was also observed; however, the reduction in pH in the presence of CA0 may have caused partial hydrolysis of its glycosidic bonds and, through the breakdown of the starch, a decrease in its viscosity. The highest viscosity of the particles that were annealed and cationized for five hours indicates the presence of extensive cross-linking within the molecule, which swelled in the presence of water. This phenomenon is confirmed by a significant increase in the viscosity of this system as the temperature rose. The accumulated thermal energy enabled the simultaneous, massive swelling of the structured grains [29]. 

In contrast, Group I demonstrated decreasing viscosity as temperature increased (Table 1). For Group IIA, similar trends were observed, whereas both Group IIB and C0 showed an increase in viscosity with rising temperature (Table 2). Generally, when mixed with water, starch forms colloidal solutions exhibiting non-Newtonian properties; thus, the relationship between its viscosity and shear rate is nonlinear and depends not only on flow conditions but also on starch structure, environmental interactions, and particle concentration.

Our study focused on comparing the temperature dependence of viscosity using the Arrhenius–Guzman equation (Equation (5)).

**Figure 3:**
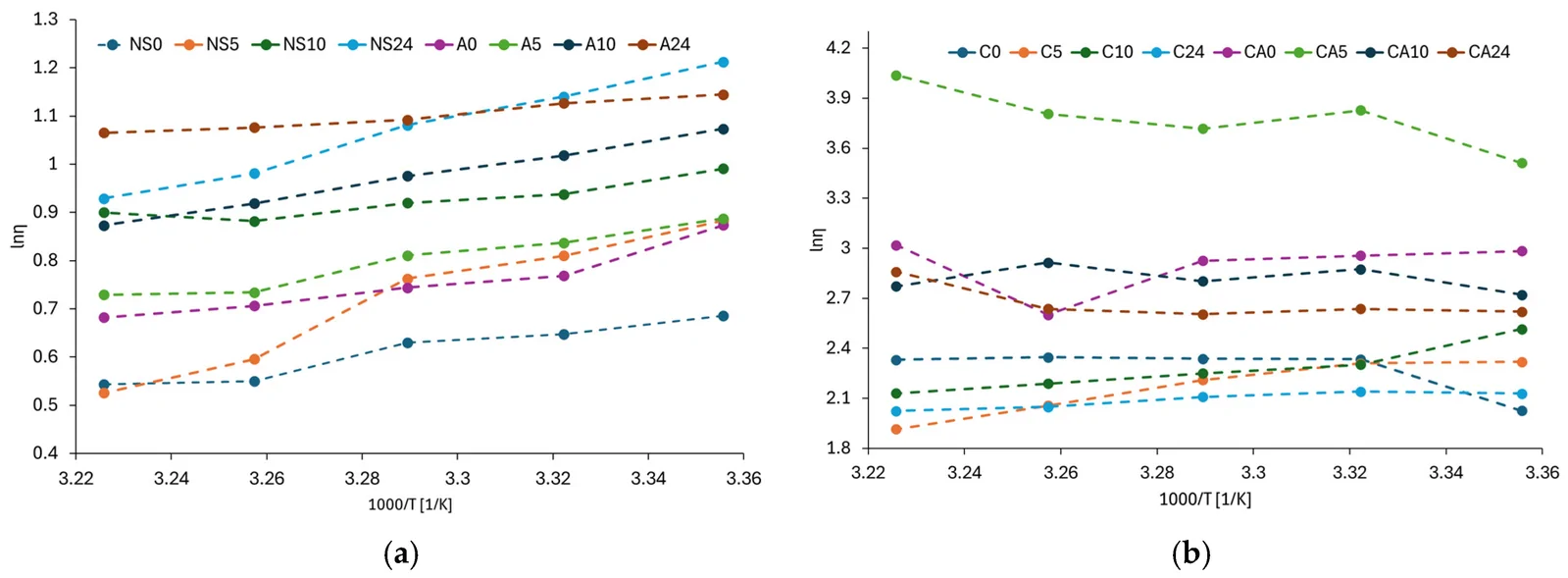
Correlation between viscosity and temperature of the Group I (**a**) and Group II (**b**) of the studied starches.

The resulting viscosity–temperature profiles differed significantly across the two starch groups. Group I exhibited markedly lower viscosity values, with viscosity decreasing as temperature increased (Figure 3a). Furthermore, increasing the degree of substitution with amino groups (ARE) resulted in higher measured viscosities (Table 1). Group II displayed greater internal variability among its subgroups. Subgroup IIA showed an inverse relationship between viscosity and temperature, with one exception: sample C0. Conversely, subgroup IIB was characterized by the highest overall viscosity values and a direct proportionality between viscosity and temperature, except for sample CA0 (Figure 3b). A dependence of viscosity on ARE was observed in both IIA and IIB, where increasing substitution correlated with increased viscosity. Crucially, this trend remained valid only within individual subgroups, rather than across all members of Group II. While sample CA5 exhibited the highest viscosity within Group II, C10 recorded a higher ARE value (Table 2). As previously established for Group II, particle size was influenced by the presence of substituted carboxyl groups. Additionally, the annealing process resulted in the deformation and flattening of starch granules. Sample CA0 yielded the highest mean particle diameter values, which may suggest stronger interactions between carboxyl groups compared to amino groups.

#### 2.1.2. pH Measurement of the Starches Tested

The pH values of the starch suspensions were measured at temperatures of 25 and 37 °C, with no significant differences observed between these two measurements. Group I displayed a pH range from 6.85 to 7.86 (Table 1), whereas Group II ranged more broadly from 4.46 to 7.18 (Table 2). Within Group I, an increase in the number of substituted amino groups (ARE) corresponded with a decrease in pH. However, annealed starches generally exhibited slightly higher pH values compared to their native counterparts. Furthermore, cationization using citrate starch (CIS) increased the initial pH; conversely, the annealing process applied to Group II samples resulted in lower pH levels relative to the corresponding non-annealed starches. This finding confirms that subjecting carboxyl groups to an annealing process enhances their reactivity.

#### 2.1.3. Starch Enzymatic Resistance Assessment

Numerous researchers have highlighted how alterations in the amorphous region of starch can significantly impact amylase activity. Generally, annealed starches derived from cereals, sago, or rice exhibit lower enzymatic resistance to α-amylases compared to their corresponding native forms [19,31]. Enzymatic degradation involves specific bonds: α-amylase hydrolyzes α-1,4-glycosidic bonds at the non-reducing end; glucoamylase hydrolyzes both α-1,4 and α-1,6-glycosidic bonds from the non-reducing end. Meanwhile, pullulanase catalyzes the hydrolysis of α-1,6-glycosidic bonds in molecules such as pullulan, amylopectin, and oligosaccharides [32]. The resistance of native starch was found to be lowest (0.11%), a value attributed to depolymerization reactions occurring at elevated temperatures [33]. In contrast, cationic starches showed higher resistance, ranging from 0.94% to 5.32%, and the incorporation of citric acid increased this range significantly, reaching 12.35% to 25.90%. Overall, the annealing process consistently resulted in a marked increase in starch resistance (Table 1 and Table 2). The values obtained for citrate starch C0 were consistent with those reported in previous studies [34]. This increased stability is mechanistically attributed to the introduction of citric acid into the starch structure, which significantly disrupts chain conformation through additional grouping and cross-link formation. These structural changes severely impede the access of amylolytic enzymes [35,36]. Furthermore, heating CIS up to 100 °C contributed further to this enhanced resistance. Consistent with these findings, other studies have documented an increase in starch resistance following thermal treatment: Kim [37] observed rising resistance as the temperature increased from 130 to 190 °C, and Lee and Tomaszewska-Ciosk reported similar increases after heating to 150 °C and 100 and 120 °C, respectively [34,35]. The cross-links formed by citric acid and high temperature create a thermally stable structure that significantly limits the formation of enzyme–substrate complexes. Furthermore, annealing creates a spatial barrier through the dense packing of helices, making it difficult for enzymes to penetrate deep into the grain. Both of these processes act synergistically, blocking enzyme activity by means of two independent structural barriers [38].

### 2.2. Analysis of the Composition and Structure of the Starches

#### 2.2.1. Fourier-Transform Infrared Spectroscopy with Attenuated Total Reflectance (FTIR-ATR) Analysis of the Starch

**Figure 4:**
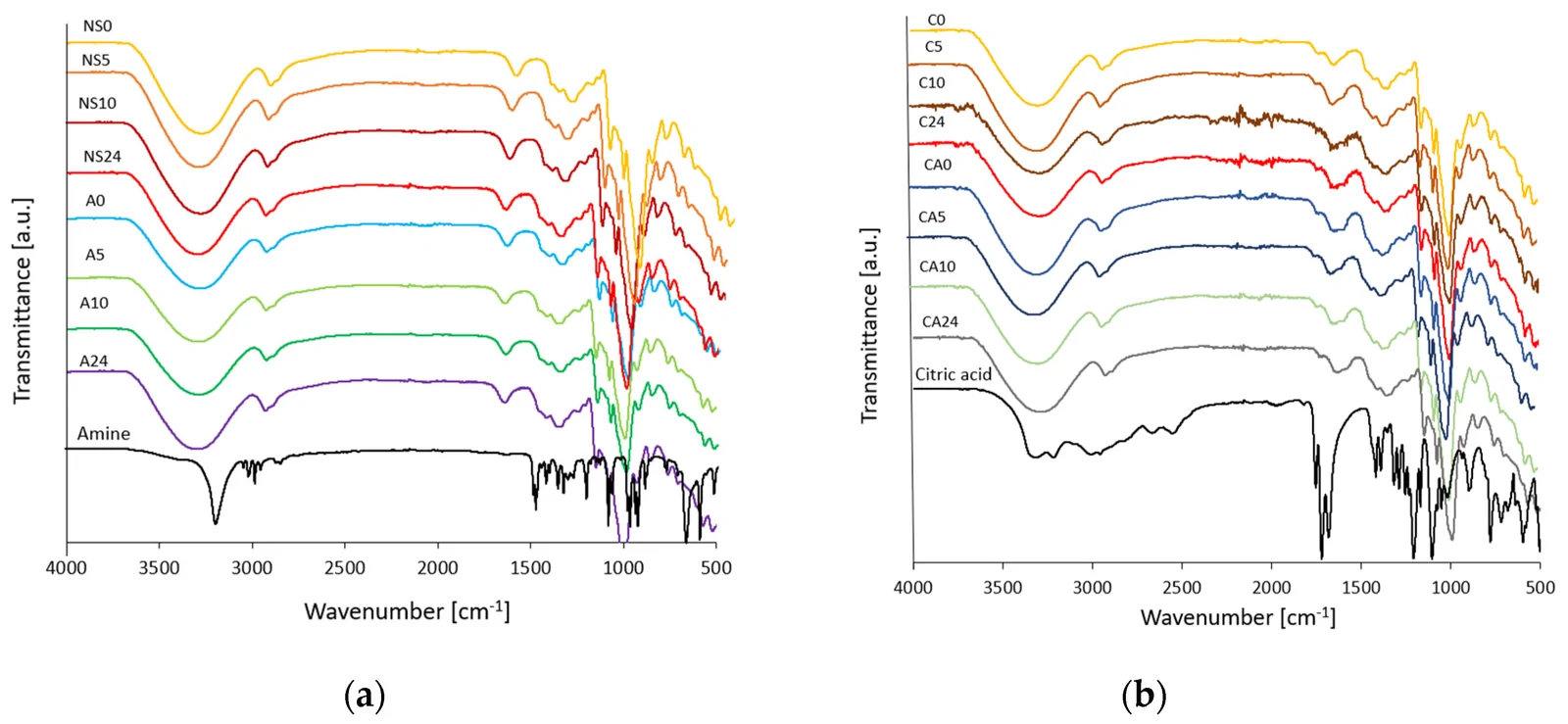
FTIR spectra of the starches of Group I (**a**) and Group II (**b**) in comparison to the precursors.

FTIR-ATR testing was performed on pure starches, utilizing amine and citric acid standards for comparative analysis to determine structural changes induced by chemical modifications. For quaternary amine samples, characteristic -C-N stretching peaks were identified in the range of 1030–1150 cm^−1^. Additionally, characteristic peaks attributed to -CH stretching vibrations appeared at 1476.72 cm^−1^ and within the range of 2950–3023 cm^−1^ [39]. The peak observed at 3091 cm^−1^ was assigned to the -OH moiety [40] (Figure 4a). Conversely, the citric acid spectra revealed -C-OH stretching vibrations at 1100 and 1210 cm^−1^, alongside -C-O bending vibrations at 1390 and 1420 cm^−1^. The peak at 1720 cm^−1^ was assigned to -C=O stretching vibrations, while the peaks at 3210 and 3320 cm^−1^ indicated the presence of the -OH moiety [41,42]. The starch spectra exhibited typical peaks for -CO bond vibrations in the range of 1000–1250 cm^−1^ and 1200–1400 cm^−1^. Furthermore, -CH bond vibrations were detected in the range of 2880–2930 cm^−1^, along with characteristic -OH absorptions at 1630 cm^−1^ and approximately 3300 cm^−1^, which are assigned to glucose (e.g., β-glycosidic linkages) [43]. In the case of CIS (Group II), an additional, noticeable peak corresponding to the -C=O vibration appeared at 1720 cm^−1^ (Figure 4b).

**Figure 5:**
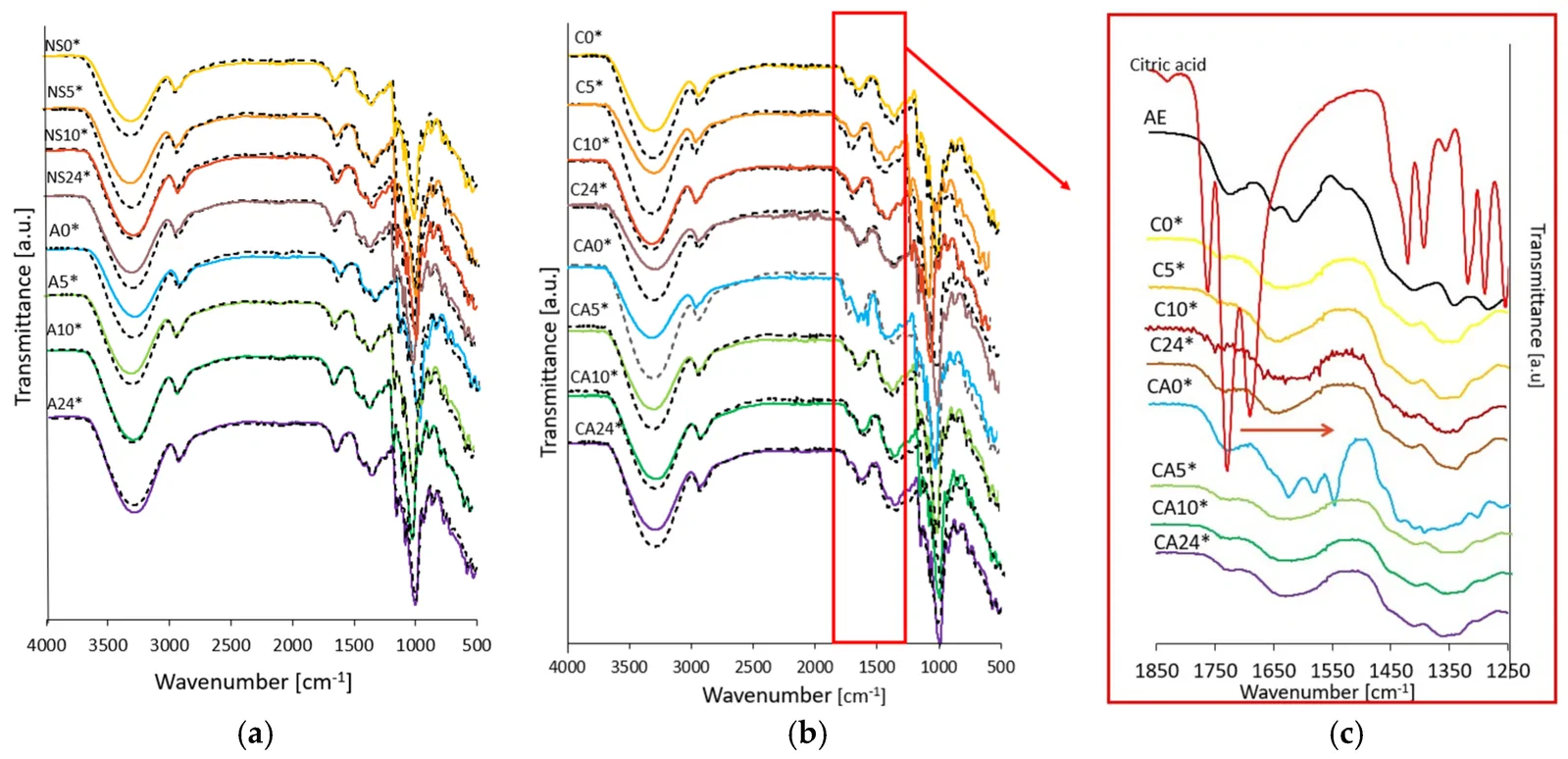
Comparison of the starches of the Group I (**a**) and the Group II (**b**) containing aronia extract after vacuum drying (the solid line) with pure starches and dry extract in the physical mixture phase (the dotted line). A star (*) shows aronia extract adding to the starches. (**c**) shows a section of the FTIR spectrum from 1250 to 1850 cm^−1^ (see the main text for an explanation); AE is vacuum-dried aronia extract.

FTIR analysis was also performed on starches containing aronia extract after vacuum drying (VAC), comparing these samples to pure starches and dry extracts mixed in a physical mixture phase (MIX). The most significant differences were observed in the broad peaks corresponding to free -OH bond vibrations at about 3300 cm^−1^. Specifically, the -OH band spectra for the VAC samples exhibited lower intensity compared to the MIX samples (Figure 5). Intensity differences were especially evident in the CIS (Group II) within the range of 1200 to 1630 cm^−1^, and also at 1720 cm^−1^ for annealed CIS (Figure 5b). These results did not indicate the formation of a strong amide bond, which would be expected from a direct reaction between carboxyl and amino groups. However, the data may suggest electrostatic interactions between the -OH groups of starch and aronia polyphenols (API), consistent with the formation of hydrogen bonds. Hishikawa et al. [44] investigated the presence of hydrogen bonds in cellulosic fibers following mercerization using FTIR spectroscopy, noting that resulting peaks in the 3300–3500 cm^−1^ range were characterized by lower absorbance. In our study, the smallest difference between VAC and MIX transmittance values at 3300 cm^−1^ was observed for NS10 and C10, while the largest was recorded for C24 and C5. Although these observations are not very precise and are subject to a number of factors that may influence the intensity of the peaks observed in this range—such as, for example, the moisture content of the samples—they merely provide information on potential relationships between starch (the carrier) and API. Further research is also required to confirm this hypothesis. Furthermore, hydrogen bonds involving a carbonyl group typically result in both a decrease in absorbance and a shift in the peak towards lower wavenumbers [45,46]. For Group II starch, mixing with aronia extract followed by vacuum drying resulted in an observable shift of peaks from the 1680–1750 cm^−1^ range to 1540–1620 cm^−1^, which is most likely attributable to the formation of hydrogen bonds between the starch carbonyl groups and API. Notably, the FTIR spectrum of CA0 with extract (CA0^∗^) revealed the largest difference and peak shift from 1720 to 1573 cm^−1^ (indicated by the red arrow in Figure 5c), which is presumed to be due to the asymmetric stretching vibration of the -C=O group [42]. TG analysis at high temperature confirmed the presence of an active carbonyl group only in the annealed citrate starch CA0 (Section 2.2.4).

#### 2.2.2. X-Ray Diffraction (XRD) Analyses of the Starches

**Figure 6:**
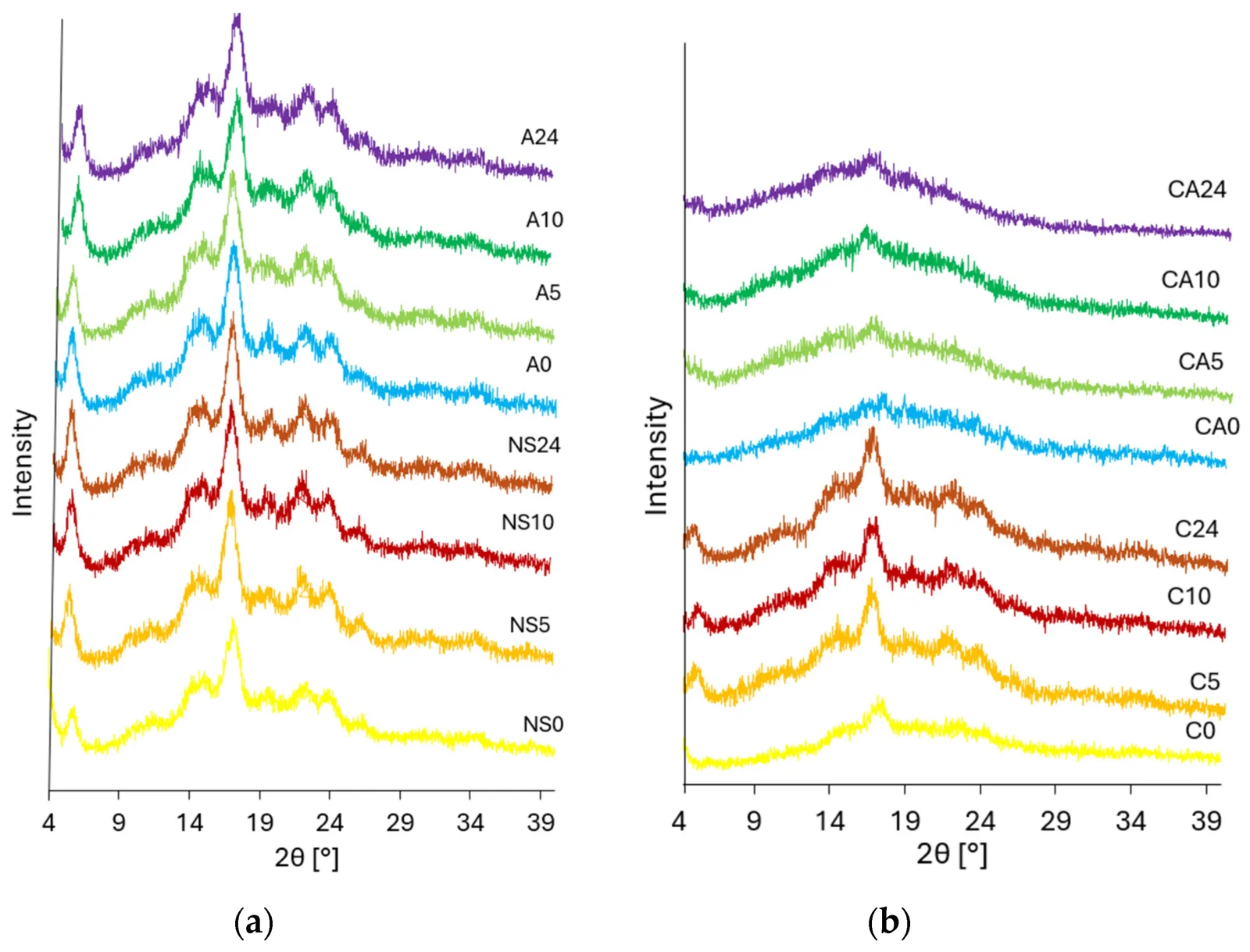
The diffractograms of the starches from Group I (**a**) and Group II (**b**).

X-ray diffraction analysis was performed on starches from both groups to assess variations in their crystallinity. The degree of relative crystallinity of the polymer is determined by analyzing the diffraction pattern, which distinguishes between ordered crystalline regions (manifested as sharp peaks) and disordered amorphous regions (appearing as broad halos). The percentage degree of crystallinity was calculated by comparing the total area under the crystalline peak to the overall scattering area (peaks + halo). As presented in the figure and table, starches from Group I and Group IIA exhibited a higher degree of crystallinity compared to those from Group IIB. Conversely, CA10 and CA0 showed the highest degrees of amorphousness. Starches belonging to both Group I and Group IIA were characterized by Type B crystallinity, exhibiting characteristic angle-of-reflection (2θ) peaks at 5.95° and 17.30°, along with a double peak at 22.39° and 23.98° [47,48]. In contrast, the starches in Group IIB were predominantly amorphous due to the combination of annealing and cationization processes. Our previous study reported that the combined action of citration and annealing yielded the most amorphous starch structure [49]. Similarly, we hypothesized that the annealing process was the major contributor to the reduction in potato starch crystallinity (Figure 6). This observation is consistent with the literature findings by Jayakoda and Hoover [19], who noted a decrease in potato starch crystallinity resulting from annealing. They attributed this decline to the disruption or reorientation of the native starch crystal structure. Furthermore, Gomes et al. [18] suggested that the reduction in crystallinity arises from interactions between amylose and amylopectin chains, leading to more compact and ordered helical packing. While Waduge et al. [50] studied barley starch and demonstrated an increase in grain size during annealing—which they explained by moisture penetration into the amorphous regions—our study also revealed a clear relationship between starch grain size and the degree of annealing (Table 1 and Table 2). Furthermore, the calculated values for the relative degree of starch crystallinity demonstrated the effect of modification on the degree of starch crystallinity (Table 3). Citrate-treated and annealed starches (Group IIB) were found to be the least crystalline (16.8–18.2%). Cationization affected the starches in the groups, and even within subgroups, in different ways. In groups IA and IIA (without annealing), it led to an increase in crystallinity, whereas in groups IB and IIB (with annealing), it mostly resulted in a decrease in starch crystallinity. The reason for this decrease in crystallinity is thought to lie in the replacement of some hydroxyl groups with larger quaternary amine groups, which may have acted as a steric hindrance, impeding the formation of an ordered structure [51]. During the cationization reaction, the amino groups penetrate mainly into the amorphous regions of the starch granule [52]. Annealing leads to an increase in amorphousness; consequently, a larger proportion of the annealed molecules undergo reorganisation during cationization. Conversely, the stabilisation of the starch’s crystallinity may have been influenced by a modification of the hydrogen-bond network [53]. 

#### 2.2.3. Differential Scanning Calorimetry (DSC) Analysis of the Starches

**Figure 7:**
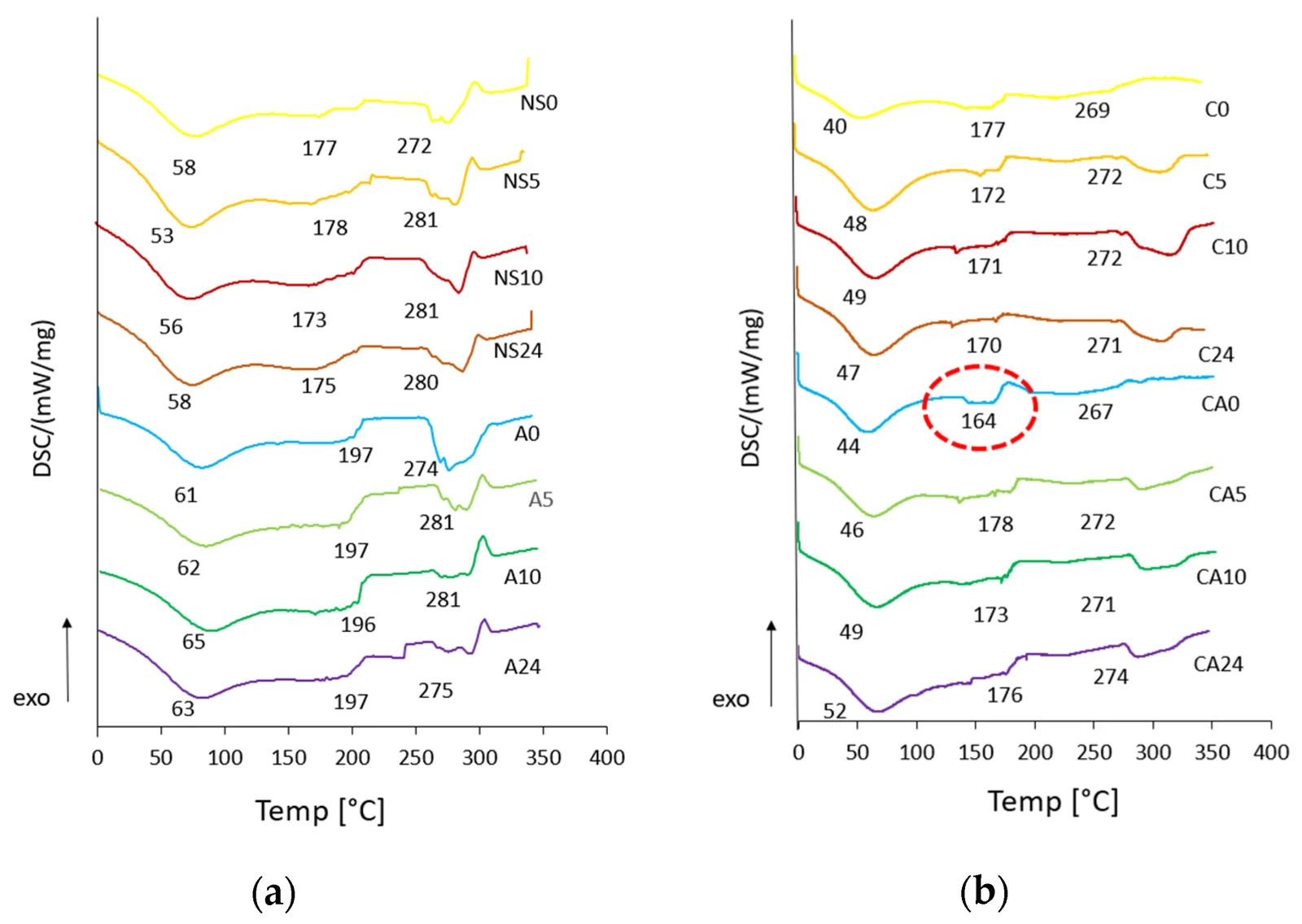
DSC thermograms of studied starches from Group I (**a**) and Group II (**b**) with glass transition temperature (T_g_), melting temperature (T_m_) and onset crystallization temperature (T_c_), respectively. The red circle indicates the citrate acid melting endothermic peak.

The DSC thermograms for all tested starches exhibited two characteristic endothermic peaks corresponding to the glass transition temperature (Tg) and melting temperature (Tm). Additionally, exothermic peaks were observed, which we designated as the onset crystallization temperature (Tc). An initial endothermic peak between 50 and 100 °C was attributed to starch dehydration [54]. The DSC results revealed that Group I samples displayed higher Tg values (53–65 °C) compared to the lower range observed in Group II (40–52 °C) (Figure 7a,b). Furthermore, annealed starches consistently showed an increase in Tg relative to their non-annealed counterparts. This elevation is attributed to annealing mobilizing polymer chains within the amorphous domains, enabling closer interaction and increased ordering between amylose and amylopectin molecules. Since these chains are more tightly packed and ordered, the resultant starch exhibits a higher gelatinization temperature [55].

While Gu et al. [56] studied the effects of citric acid-assisted annealing combined with heat-moisture treatment at 110 °C on lotus starch—observing both a decrease (compromising thermal stability) and an increase (improving thermal stability) in gelatinization temperature, respectively—Fonseca et al. [57] suggested that the increase in Tg during annealing might be due to changes in the melting temperature of crystalline lamellae. This is attributed to the breakdown and subsequent reorganization of less stable crystallites; consequently, amylose linkage chains or amylopectin B chains adopt a more energetically favorable conformation after annealing, which has a lesser effect on the overall melting temperature of the crystalline lamellae [58].

We utilized several secondary parameters:

1. The ratio r = Tg/Tm: Philips [59] stated that Tg can be related to coordination number in a bonded network, accounting for intermolecular covalent and hydrogen bonds. He proposed that the ratio r = Tg/Tm serves as an indicator of the degree of clustering or mesoscale order in glassy materials; thus, a higher r value signifies greater clustering. He found r = 0.52 for disaccharide sucrose, which has a crystallinity of 95 to 100% but asymmetric rings that suppress clustering. In our study, Group II exhibited lower r parameters (0.23–0.30) compared to Group I starches (0.30–0.33) (Table 3). These values show a stronger correlation with the degree of starch crystallinity, with Group I exhibiting a higher degree of crystallinity than Group II;
2. Thermal Stability (ΔT = Tg − Tc): In our previous work, we identified the difference between Tg and Tc (ΔT) as a key parameter determining the thermal stability of the investigated starches; a higher ΔT value indicates greater thermal stability [49]. The present study recorded ΔT values ranging from 212 to 229 °C, with sample A24 having the lowest and C0 exhibiting the highest (Table 3). In this case, the cationization of native and annealed starch resulted in an increase in the thermal stability of the molecules. In contrast, the cationization of citrate starch led to a decrease in its thermal stability (with the exception of CA5). Cationic substituents are characterized by lower thermal stability than hydroxyl groups. During heating, they are the first to degrade. The higher the degree of substitution with amino groups, the greater the degradation [60]. An increase in the thermal stability of starch was observed in cases of low substitution with cationic groups, which increased the mobility of the polymer chains, resulting in a tighter, more ordered “packing” [61]. The annealing process resulted in a decrease in the thermal stability of the starch. The reorganization of the molecular structure led to greater susceptibility to thermal energy and faster degradation of the carbohydrate backbone.

Endothermic temperatures in the range of 140–320 °C caused the starch to break down [54]. The starches from Group IA and Group II, with the exception of CA0, exhibited a melting temperature (Tm) ranging from 170–178 °C. In the absence or small amount of water, the crystalline regions of starch granules undergo a melting transition at approximately 150–170 °C. Annealed starches are more durable and melt at higher temperatures than native ones [62]. In our study, the Tm of annealed Group I starches had increased by 20 °C, while for Group II, the increase was only a few degrees. It might be suggested that the carboxyl moieties of CIS as well as amine groups may have affected the annealing impact on the Group IIB starches. The endothermic peak of the CA0 attendance at 164 °C may support this phenomenon. This is likely responsible for the breakdown of the carboxyl groups in the starch citrate [63,64]. Shi et al. [64] compared the endothermic melting peaks of starch citrate containing glycerol, which occurred around 150 °C, with the melting point of anhydrous citric acid, which was 159 °C. This peak was observed only for non-cationic annealed starch citrate, as confirmed by TG analysis.

#### 2.2.4. Thermogravimetric (TG) Analysis of Starches

**Figure 8:**
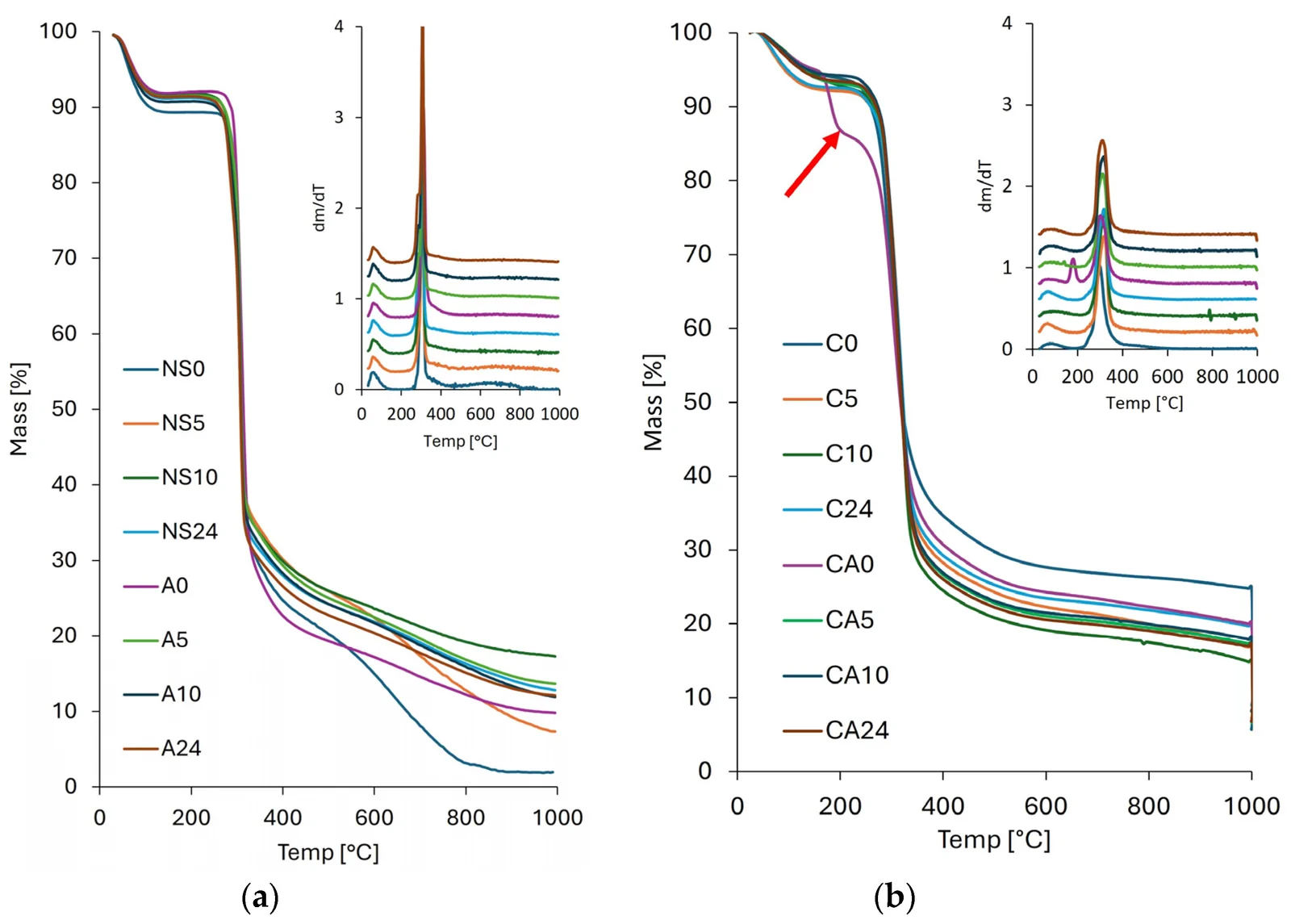
TG thermograms of the studied starches from Group I (**a**) and Group II (**b**) with appropriate derivatives (the right corner). The arrow indicates the breakdown of carboxyl groups.

The TG thermograms for all starches are shown in Figure 8. Group I starches exhibited a mass loss of 10% at 110 °C. Subsequently, the initial temperature for mass loss resumed, ranging from 260 °C to 280 °C, and concluded at 315–325 °C, respectively. The greatest weight loss was observed for NS0 (98%), and the smallest for NS10 (83%) (Figure 8a). Group II reached a weight loss of up to 4% at 110 °C (C5, C24). For most starches, the initial temperature of this renewed weight loss was 240 °C, and it ended in the range of 325–350 °C. The greatest weight loss was recorded for CA24 (85%), while the smallest was for C0 (75%) (Figure 8b). A three-stage weight loss pattern was observed for CA0. The onset temperatures for the second and third stages of weight loss were 165 °C and 250 °C, respectively. TG measurements confirmed the breakdown of carboxyl groups in CA0. According to Shi et al. [64], this second decrease is directly linked to the interaction between citric acid and starch, which forms bonds between them. This hypothesis is supported by the presence of hydrogen bonds at the CA0 carboxyl groups, as revealed by FTIR analysis. A comparison of the residual starch masses across both groups showed lower thermal stability in Group I compared to Group II. C0 exhibited the highest thermal stability, whereas NS0 showed the lowest. Furthermore, a relationship was observed between annealing and thermal stability within Group I; annealing increased the thermal stability of these starches. No such relationship was evident for Group II starches. The enthalpies of starch gelatinization ranged from 209.8 to 396.2 J/g. A direct relationship can be observed between enthalpy and the degree of crystallinity of the particles (Table 3). Specifically, higher crystallinity in the starch requires more energy in the form of heat (ΔH) to break the ordered hydrogen bonds of amylopectin [65].

#### 2.2.5. Scanning Electron Microscopy (SEM) Evaluation of Starches

**Figure 9:**
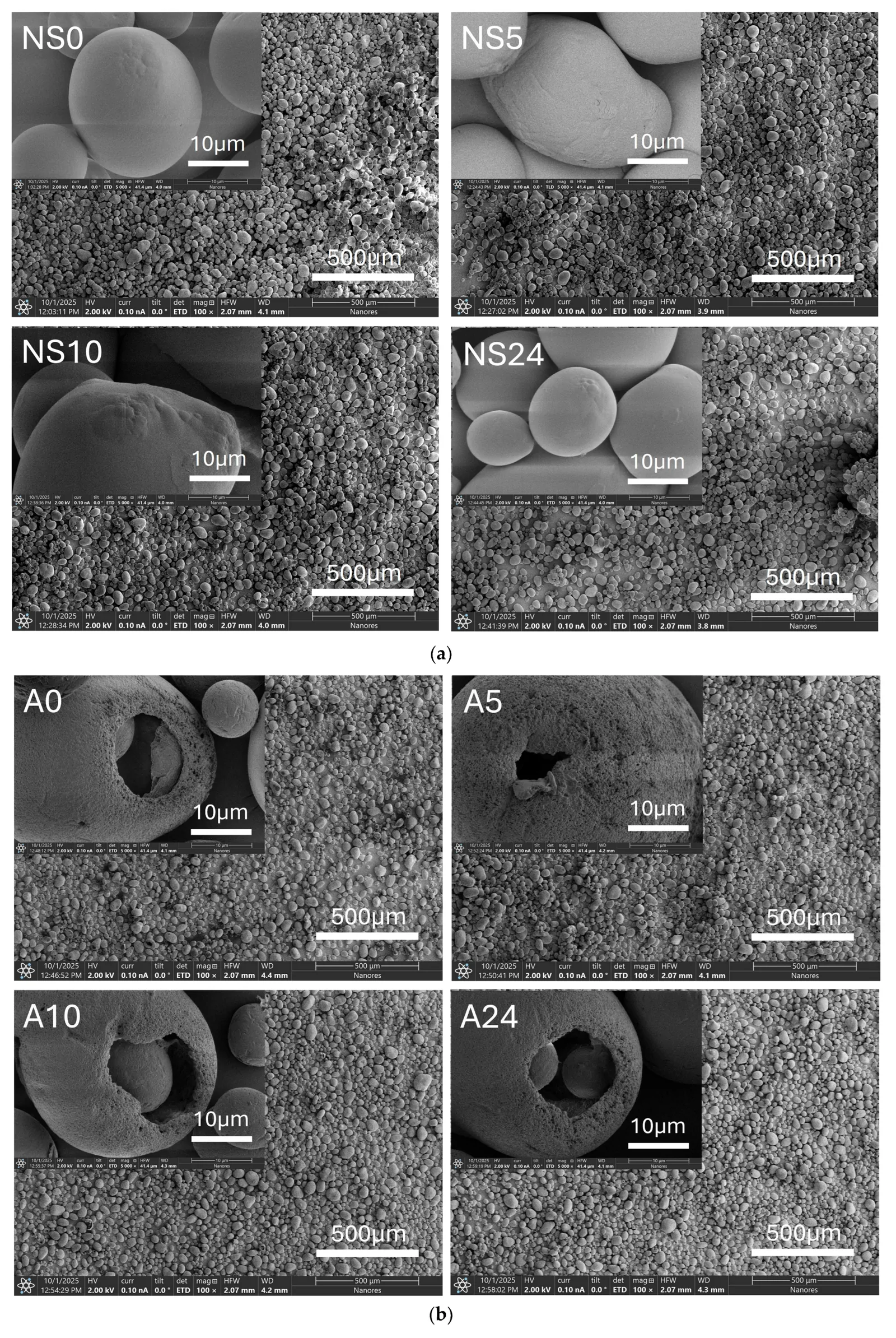

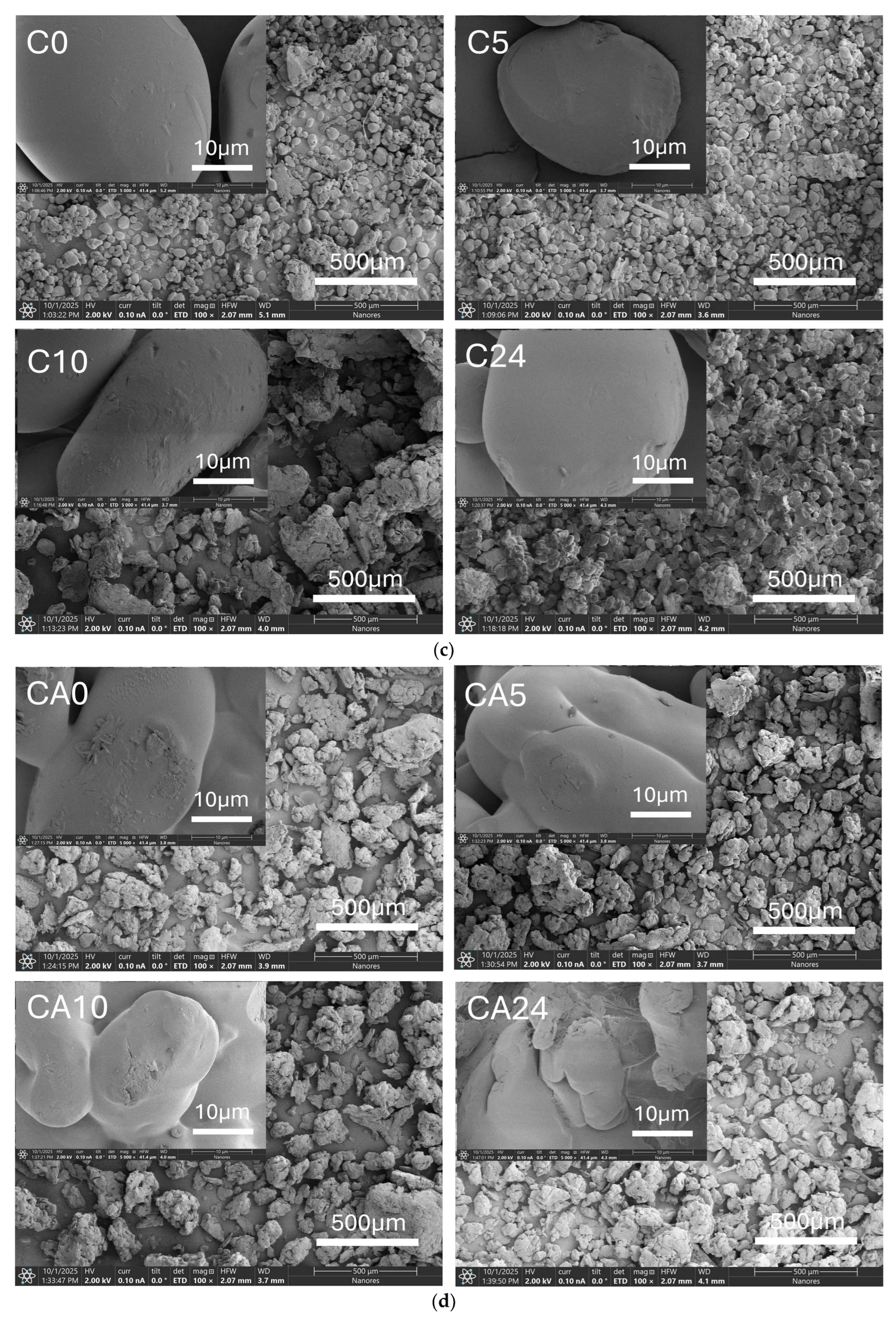
The images of SEM magnitude of the starches from the Groups: IA (**a**), IB (**b**), IIA (**c**) and IIB (**d**).

Microscopic images of the starch granules were captured at magnifications of 100× and 5000×. The starches exhibited characteristic morphologies consistent within each group. Group IA starches were characterized by rounded shapes, smooth surfaces, and diameters ranging from 10 to 30 μm (Figure 9a). Annealed starches from Group IB appeared spherical but featured visible grooves, perforations, and holes, with diameters similar to those of Group IA (Figure 9b). Starches from Group IIA were characterized by a more irregular shape, visible surface irregularities, and the presence of agglomerates. Their diameters were notably larger, starting at 30 μm (Figure 9c). The starches in Group IIB exhibited the most irregular structure, numerous folds, and contained the highest density and largest agglomerates (Figure 9d).

The formation of hollows in Group IB starch is likely attributable to annealing as a heat-moisture modification. Li et al. [66] studied lily starch treated with both heat-moisture at 110 °C and acidification processes. Similarly, Deng et al. [67] used microwave heat alongside heat-moisture treatment at 90 °C on potato starch. Both studies observed a hollow structure in the hilum region of the annealed starch, but no such changes were visible in the acidified starch. They suggested that the hilum region is located within the relatively fragile amorphous zone. In our study, however, the annealed starch without acidification displayed additional pores and perforations, which could also contribute to increased fragility.

The irregular and bumpy surface texture and increased size observed in Group II starches may be primarily caused by acidification. Kaur et al. [68] studied the physicochemical and morphological properties of acid-treated starches from banana, sweet potato, lotus stem, and wheat. They reported that acidification increased the size of banana and lotus starches, while SEM photographs showed that all types developed irregular shapes and a tendency to agglomerate, particularly sweet potato and wheat starches. Liu et al. [69] studied the morphology of esterified waxy maize starch with citric acid. Previous studies have consistently reported an increase in particle size accompanied by the development of rougher and more eroded surface morphology following the substitution of hydroxyl groups in starch with ester functionalities. Similar observations were made in the present study: as the degree of modification increased, the starch granules exhibited progressively greater surface roughness, structural deformation, and a stronger tendency toward agglomeration. These morphological changes are likely due to the introduction of functional groups capable of intermolecular interactions, especially carboxyl and amino moieties. The presence of these groups probably enhanced hydrogen bonding, electrostatic interactions, and molecular bridging between adjacent granules, thereby promoting aggregation and altering the surface architecture. Furthermore, the observed effects were intensified after heat-moisture treatment, suggesting that thermal processing facilitated molecular rearrangement and strengthened the interactions among the incorporated functional groups. Consequently, the combined effect of chemical modification and heat-moisture treatment resulted in more pronounced structural disruption and aggregation of the starch particles.

### 2.3. Release of Chlorogenic Acid (CHA) from Starch Tablets 

**Figure 10:**
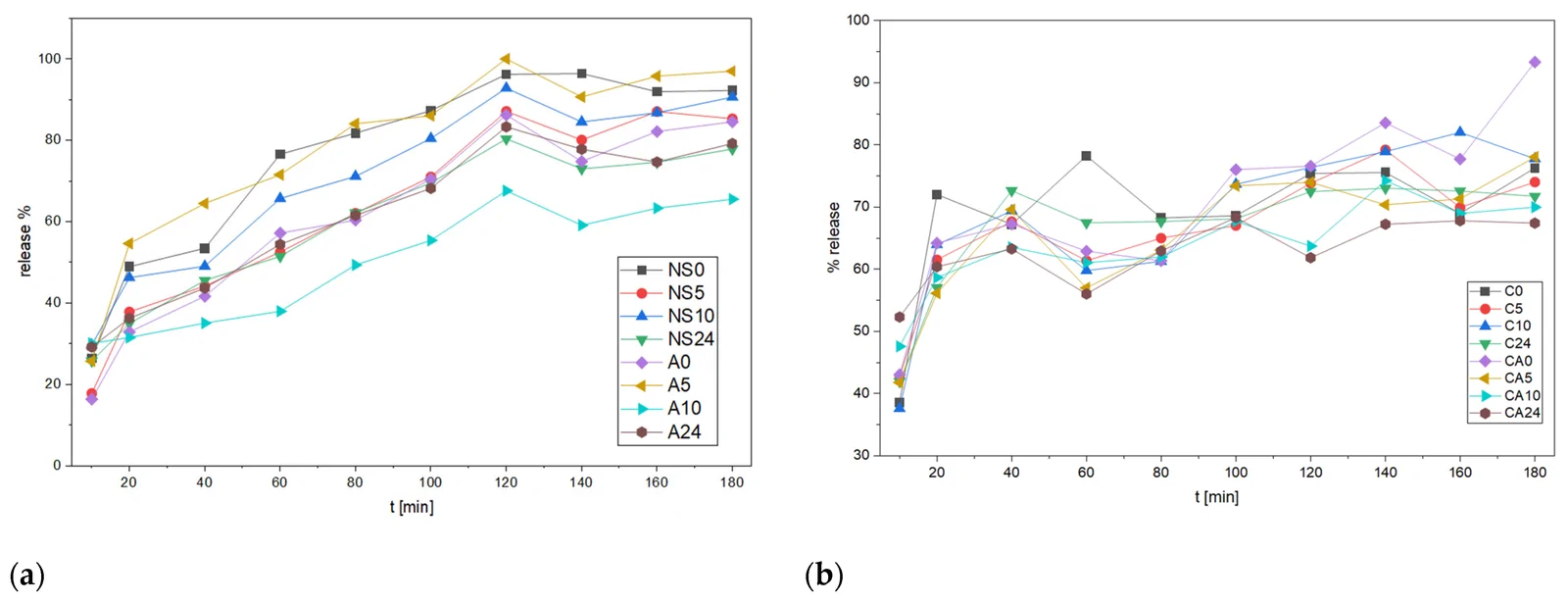
The chlorogenic acid release from the Groups: I (**a**) and II (**b**) of starches.

Figure 10 illustrates the release kinetics of CHA from the chokeberry extract in starch tablets. After three hours, Group I starches showed higher levels of released CHA (80–100%) (Figure 10a) compared to Group II starches (70–90%) (Figure 10b), with the exception of A10. Generally, the annealing process appeared to decrease the percentage of released CHA. Furthermore, applying more starch modifications resulted in a more complex or varied release profile.

**Figure 11:**
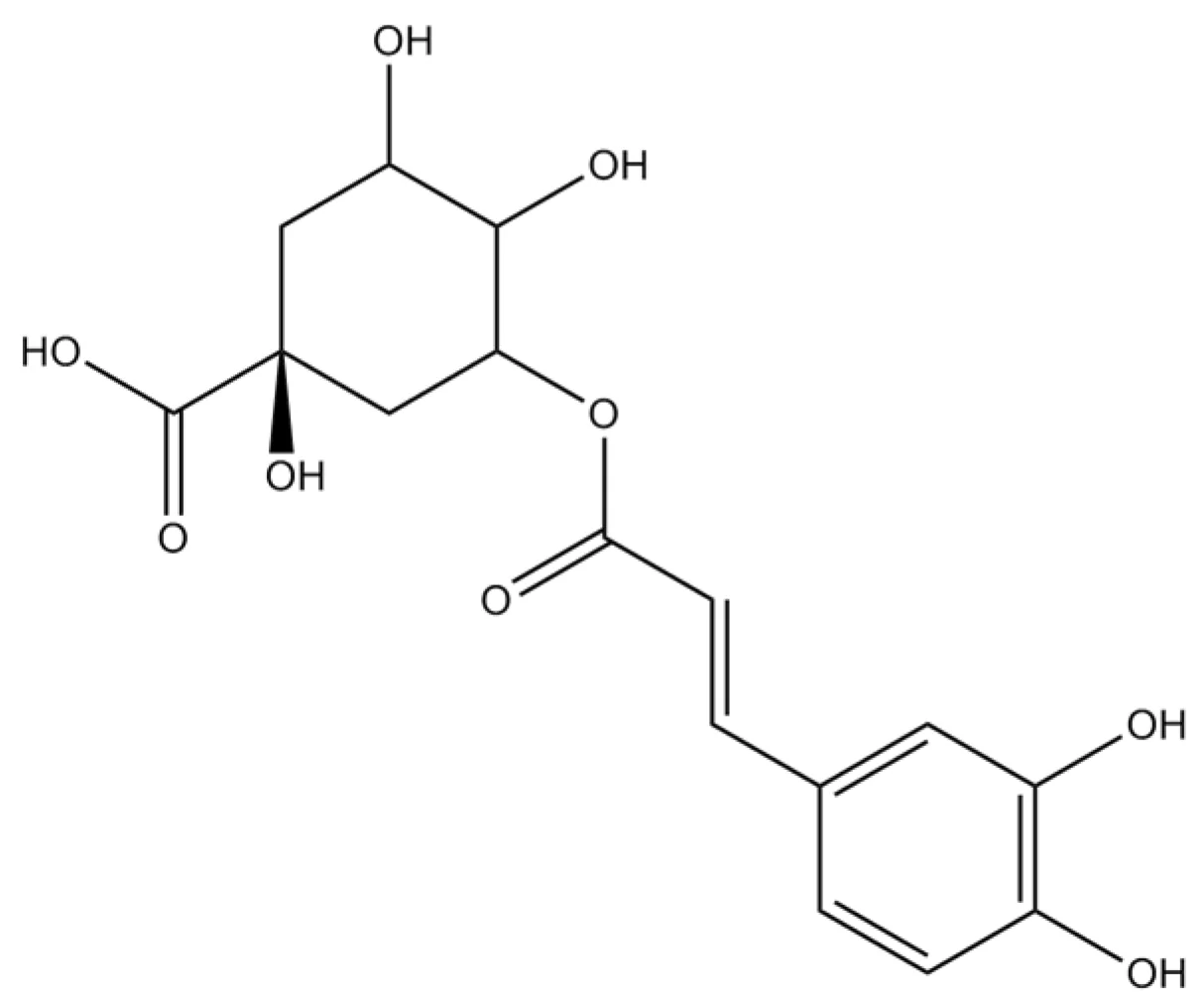
Chlorogenic acid (CHA) structure.

The lower percentage of CHA released in Group II correlates with the FTIR peaks observed for starches containing aronia extract (AE), which showed greater variations in the intensity of the -OH group peaks. This difference likely indicates that hydrogen bonds formed between AE and the carboxyl groups of CHA (Figure 11). Conversely, the weakest correlation was observed between CA0 and CHA, as the degree of CHA released from CA0 was the highest among Group II starches (Figure 10b). This suggests that CA0 may have formed hydrogen bonds with another component present in AE that possesses positively charged groups, rather than bonding with the negatively charged carboxyl group of CHA (Figure 11), or alternatively, that there is a greater affinity for forming intermolecular hydrogen bonds within the CA0 molecules themselves. The Group II starches substituted with amino groups exhibited a lower degree of CHA release compared to their unsubstituted counterparts (C0 and CA0). Overall, the release results were consistent with the appropriate ARE values calculated for these starches (Table 1).

The release kinetics of CHA from starch were analyzed using various models, assessed based on the linear regression coefficient r^2^ (Table A1). Generally, the CHA release from Group I starches was most frequently described by the Weibull or Korsmeyer–Peppas models. An exception was A10 starch, whose release profile was best described by a first-order kinetic model. For the Weibull model, the parameters *α* and *β* are critical. The parameter *α* corresponds to the time scale of the process and determines the rate of active substance release; consequently, a smaller α indicates a faster attainment of the plateau phase [70]. The parameter *β*, in turn, characterizes the release mechanism: a value of *β* ≤ 0.75 suggests diffusion following Fick’s first law; 0.75 < *β* < 1.0 describes diffusion combined with Type II transport; and values of *β* ≥ 1.0 indicate Type II transport [70,71]. In our study, Group I starches followed the Weibull model: A0 exhibited the smallest value for *α* (0.0299), while NS24 showed the largest (0.0819). Regarding *β*, this group displayed values below 0.75 for NS10, NS24, and A24, but values within the 0.75−1.0 range for NS0, NS5, A0, and A5. This suggests the involvement of factors beyond the concentration gradient that may have accelerated CHA release, such as solubility or interaction with the external environment. The release from A24 best fit the Korsmeyer–Peppas model, which is typically used to describe controlled release from an expandable polymer matrix [72], though this mechanism applies up to 60% content release [73]. The release from A10, modeled by a first-order kinetic equation, indicates a relationship between particle dissolution and surface interactions [74]. Group II starches followed the first-order kinetic model. Conversely, C0 was best fitted by the Korsmeyer–Peppas model. This suggests that interactions between the starch particle surface and the surrounding environment significantly influenced the release. These findings are further supported by the structural characteristics of individual starch particles observed in the SEM images (Section 2.2.5).

Given the relatively poor fit between Group II release and the selected kinetic models, it is worth highlighting the possible reasons for this phenomenon. A first-order model refers to a system in which the rate of drug release is solely a function of the remaining drug concentration. This model often has poor physical interpretation in complex polymeric systems. It frequently provides a good statistical fit simply as a result of the concentration gradient decreasing over time (and the resulting decrease in the release rate), rather than because it reflects a specific molecular mechanism [75]. Korsmeyer–Peppas model is subject to strict mathematical limitations. As already mentioned, it is physically meaningful only during the initial phase of release (typically the first 60% of the fractional release) and cannot be accurately applied to the entire release curve. Furthermore, when analysing the diffusion release exponent (*n*), this model often fails to provide accurate results for complex systems involving multiple mechanisms, such as those in which swelling and intense erosion occur simultaneously [76]. Unfortunately, the studies on citrate-induced starch release using the above models may not have fully replicated the process due to starch swelling and its tendency to aggregate. Another approach could include different models, such as the Peppas–Sahlin model, which accounts for swelling and erosion [77], or the Hopfenberg model, which takes into account the geometry of the system and surface erosion [78]; however, the study was not concentrated on erosion experiments.

## 3. Materials and Methods

### 3.1. Materials

The following reagents were utilized in this study: native potato starch NS0 ( molecular weight about 15 × 10^5^ g/mol; molecular weight of a mer 162.14 g/mol, moisture max. 20.0%, pH 5.5–8.0, viscosity min. 1100 [Brabender Units, BU], 80 g carbohydrate per 100 g) (PEPEES S.A., Łomża, Poland); (3-chloro-2-hydroxypropyl)trimethylammonium chloride solution (60 wt.% in H_2_O) (Sigma-Aldrich, Darmstadt, Germany); sodium hydroxide (Stanlab, Lublin, Poland); and citric acid monohydrate (pH 2.36, extract 50%) (Stanlab, Lublin, Poland). The *Aronia melanocarpa* fruits were picked on September 2024 from chokeberry bushes (Krapkowice County, Poland).

### 3.2. Starch Modification Procedures

#### 3.2.1. Hydrothermal Modification (Starch Annealing, ANN)

Native potato starch (NS0) was immersed in distilled water and stirred at 320 r.p.m. for 24 h at 58 °C. Following the treatment, the starch slurry was dried in an air oven at 30 °C before any further chemical modification steps were performed.

#### 3.2.2. Chemical Modifications

##### Starch Etherification (Cationization)

**Figure 12:**
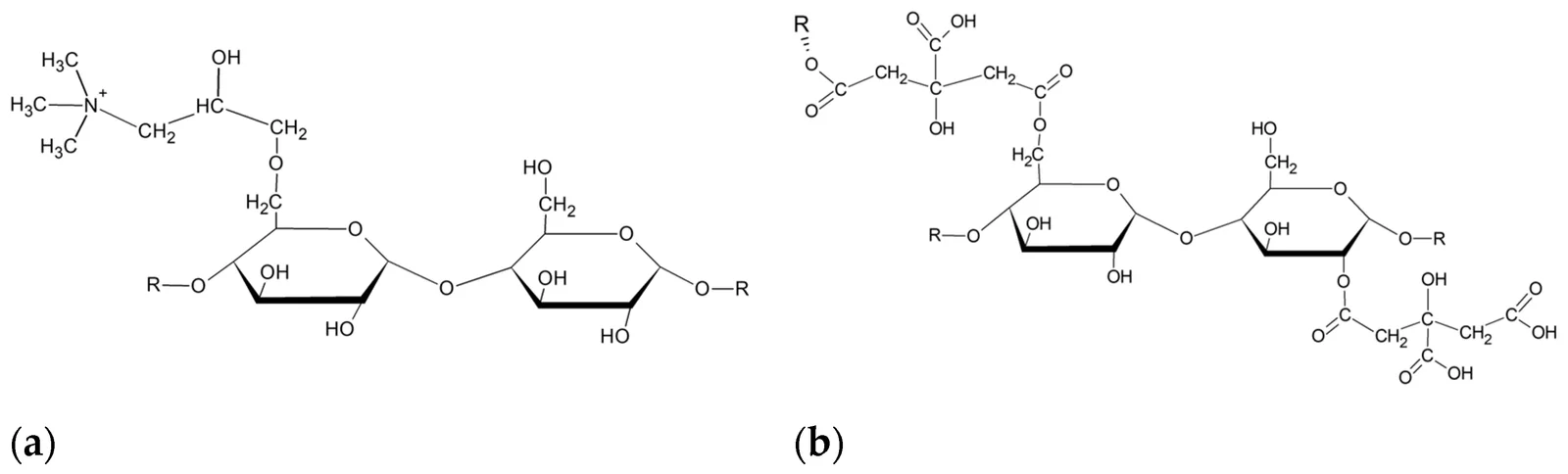
The structural models of cationic starch (CAS) (**a**) and citrate starch (CIS) (**b**); R refers to a glucose.

**Figure 13:**
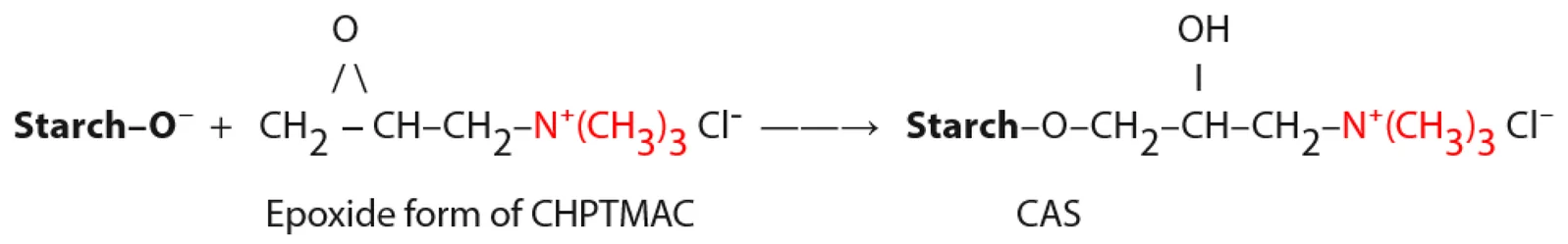
A schematic diagram of the starch cationization process [80]. Amino groups are highlighted in red.

Suspensions of either native starch (NS0) or annealed starch (A0) were prepared. (3-chloro-2-hydroxypropyl)trimethylammonium chloride(3-chloro-2-hydroxypropyl)trimethylammonium chloride (CHPTMAC) and 3% sodium hydroxide solution were added simultaneously to the mixtures. The resulting mixtures were stirred at 420 r.p.m. for 5, 10, or 24 h, depending on the modification group. Subsequently, the suspensions were washed with 95% ethanol, rinsed six times with distilled water, and acidified using 7.5% HCl until a pH of 3.5 was achieved. The resulting cationic starch (CAS) (Figure 12a) was then dried in an air oven at 30 °C for 12 h [79]. This procedure is schematically represented below, starting from the epoxy form of CHPTMAC (Figure 13).

##### Starch Esterification/Cross-Linking

Distilled water was added to either native starch (NS0) or annealed starch (A0). Citric acid was subsequently added to the starch at a concentration of 10% *w*/*w* relative to the dry starch and thoroughly mixed. The mixtures were then air-dried at 30 °C for 12 h and roasted at 100 °C for 3 h. They were washed with 95% ethanol and subsequently rinsed 30 times with distilled water. The resulting citrate starch (CIS) (Figure 12b) was dried in an air dryer at 30 °C for another 12 h. Some of the CIS was subjected to further cationization modifications in accordance with the formula in Section Starch Etherification (Cationization).

Various combinations of starch types were prepared following these procedures and are detailed in the groups listed in Table 4.

### 3.3. Incorporating Chokeberry Fruit Extraction into the Starch

A chokeberry fruit extract was prepared for both Fourier-Transform Infrared (FTIR) analyses and release tests. One hundred grams of frozen chokeberries were ground and immersed in 330 mL of acid ethanol (0.1 M HCl/96% ethanol, 5/95 *v*/*v*) for one hour. The aronia extract (AE) was then filtered through a soft quality filter at 25 °C. This AE was dripped onto the pure starch powder (2:1 *w*/*w* ratio) and conditioned for one hour. Both the AE and the starch soaked in AE were subsequently vacuum-dried for 24 h.

### 3.4. Determination of Physicochemical Starch Properties

#### 3.4.1. Determination of Amination Reaction Efficiency (ARE) of the Starches

The percentage of nitrogen (*N*) in the dry starch mass was determined using the Kjeldahl method [81]. Approximately 0.5 g of the dry starch was hydrolyzed with 25 mL of concentrated sulfuric acid (H_2_SO_4_) at 370 °C for two hours. After cooling, distilled water was added, and titration using sodium hydroxide was performed. The nitrogen substitution (*NST*) was calculated for native starch (Group I) and citrate starch (Group II) as:

(1) $$N{ST}_{I}=\frac{162N}{1400-\left(181.5N\right)}\;\left[\%\right]$$

(2) $$N{ST}_{II}=\frac{706N}{1400-\left(181.5N\right)}\;\left[\%\right]$$

respectively, where *N* is the percentage nitrogen content in the dry starch. 

The amination reaction efficiency (*ARE*) was calculated as follows [82]:

(3) $$ARE=\frac{NST\cdot 100\%}{\frac{amine}{starch}\;molar\;ratio}\;[\%]$$

#### 3.4.2. Determination of Hydrodynamic Diameter D[4,3] of the Starches

To examine the swelling properties of the starch, the particle size distribution was determined. Laser diffraction using a Malvern Mastersizer 2000 (Malvern Instruments Ltd., Worcestershire, UK) was utilized to measure the volume diameter of the starch in aqueous solution at 25 ± 0.5 °C. The analyzed suspensions were treated with ultrasound (20 Hz/10 s) before and after each measurement to disrupt starch agglomeration. Temperature conditioning was performed for 10 min, and the average volumetric diameter D[4,3] of the particles was subsequently determined.

#### 3.4.3. Determination of pH of the Starches

The pH values of aqueous starch suspension samples were measured using a potentiometric method, employing a pH meter (CPC-505, Elmetron, Zabrze, Poland) and an ERH-11S electrode (Hydromet, Gliwice, Poland). Starch suspensions with a concentration of 10% *w*/*w* were tested. All pH measurements were performed at two distinct temperatures: 25 ± 1 °C and 37 ± 1 °C. Each measurement was taken in triplicate (three times).

#### 3.4.4. Determination of Viscosity of the Starches

The viscosity of the 10% (*w*/*w*) starch suspensions was measured using a Brookfield KF40 Höppler viscometer (Ametek Brookfield, Middleboro, MA, USA). The falling time of the steel ball was recorded at temperatures of 25, 28, 31, 34 and 37 °C at an angle of 60°. Each measurement was taken in quadruplicate (four times).

The dynamic viscosity (*η*) was calculated using the following equation:

(4) $$\eta =t\left(\rho_{1}-\rho_{2}\right)KF$$

where *η* is dynamic viscosity [mPa·s], *t* is the flow time of the steel ball [s], *ρ*_1_ is the density of the steel ball [g/cm^3^], *ρ*_2_ is the density of the samples [g/cm^3^], *K* is a ball constant [mPa·cm^3^/g], and *F* is a working angle constant. 

The dependence of viscosity on temperature was subsequently expressed by the Arrhenius–Guzman equation [83]:

(5) $$ln\eta =\ln\left(k\gamma^{n-1}\right)+\frac{\Delta G}{RT}$$

where *γ* is shear rate, and *k* is a constant, Δ*G* is the activation energy, *R* is the gas constant, and *T* is the temperature.

#### 3.4.5. Determination of the Resistance of Starch Preparations to Amyloglucosidase

In conical flasks, 38 g of a 0.72% suspension of the starch preparations in water was prepared. This mixture was brought to a boil and maintained at this temperature for five minutes. Following heat treatment, the sample was cooled to room temperature, and any weight loss due to water evaporation was replenished back to the initial value (38 g). Subsequently, 34 mL of acetate buffer (pH 4.35) was added. The resulting mixture was then incubated in a water bath at 37 °C with continuous stirring. Enzymatic hydrolysis was initiated by adding 4 mL of Dextrozyme DX 1.5 enzyme preparation (NOVONESIS, Lyngby, Denmark), which contains glucoamylase, α-amylase, and pullulanase. The amount of enzyme was selected to ensure complete saccharification of native starch within two hours. Samples of the hydrolysate (1 mL) were collected at one-hour intervals and subsequently centrifuged (5000 r.p.m., 5 min). Hydrolysis completion was defined as the point when the absorbance values obtained in three consecutive measurements remained unchanged. From the resulting supernatant, 10 μL of the solution was taken and transferred to a cuvette containing 1 mL of BIOSYSTEM reagent (Barcelona, Spain), which contains glucose oxidase and peroxidase. The mixture was then thoroughly mixed and incubated at room temperature for 15 min. Absorbance was measured at a wavelength of λ = 500 nm using a CECIL 2000 colorimeter (Villenave d’Ornon, France). Measurements were performed against a blank sample, which consisted solely of the reagent with acetate buffer. The glucose content was then determined by reading from a previously prepared calibration curve. 

The enzymatic resistance of the starch preparations (*ER*) was calculated using the equation:

(6) $$ER=100-\frac{100x}{0.396}[\%]$$

where *ER* is the enzymatic resistance of the starch preparations [g/100 g], *x* is a glucose content read from the standard curve [mg], and 0.396 is the maximum amount of glucose produced from 0.36% natural starch paste [mg].

### 3.5. Determination of the Composition and Structure of the Starches Under Investigation

#### 3.5.1. Determination of FTIR-ATR Analyses of the Starches

Fourier-Transform Infrared (FTIR) analysis was conducted using an FTIR spectrophotometer equipped with a Fourier transformation (FTIR) and Attenuated Total Reflectance (ATR) accessory (Nicolet 380, Thermo Scientific, Waltham, MA, USA), utilizing OMNIC software (OMNIC 9.3.30, driver version: 9.2, firmware version: 1.00). Samples of pure starches, the vacuum powder of starches combined with AE, and physical mixtures of AE and starch (1:10 *w*/*w*) were prepared by grinding them into a fine powder using an agate mortar. The spectra of these powders were recorded over a wavelength range of 400 cm^−1^ to 4000 cm^−1^, collecting 32 scans per sample with a resolution of 4 cm^−1^. Data analysis was performed using dedicated computer software (OMNIC Spectra 2.0).

#### 3.5.2. Determination of XRD Analyses of the Starches

Powder XRD (PXRD) data were recorded on a Bruker D2 PHASER diffractometer (Bruker AXS, Karlsruhe, Germany) equipped with a Lynxeye detector and utilizing Cu Kα radiation (1.5418 Å). Samples of the pure starches were ground into a powder using an agate mortar. The measurements were performed at 295 K with a slit size of 0.2 mm and a shutter opening of 1.0 mm. Diffractograms were obtained between 2.5° and 40° (2θ) (step size of 0.02° (2θ) and 0.5 s/step). The X-ray generator operated at 30 kV and 10 mA. The resulting PXRD patterns were processed using the software Diffrac.Eva V 3.2 (Bruker AXS). The degree of crystallinity was calculated from the ratio of the area of the crystalline peaks to the total signal.

#### 3.5.3. Determination of DSC Analyses of the Starches

Differential scanning calorimetry (DSC 214, Polyma, Netzsch, Selb, Germany), using nitrogen as the cooling gas, was employed. To determine the plasticizing effect of absorbed water, a heating cycle was executed from 0 °C to 350 °C at a rate of 5 K/min. Samples consisting of 3–5 mg of pure starches were ground into a powder using an agate mortar and analyzed in aluminum pans fitted with perforated lids. The obtained DSC curves were analyzed using applicable software (Proteus, Netzsch 8.0.3, Selb, Germany).

#### 3.5.4. Determination of TG Analyses of the Starches

Thermogravimetric analysis (TG 209 F1, Libra^®^, Netzsch, Selb, Germany), using nitrogen as the cooling gas, was performed. For determining mass loss processes, a heating cycle was applied from 25 °C to 1000 °C at a rate of 10 K/min. Samples consisting of 5–6 mg of pure starches were ground into a powder using an agate mortar and placed in a corundum crucible. The obtained TG curves were analyzed using applicable software (Proteus, Netzsch 8.0.3).

#### 3.5.5. The Scanning Electron Microscope (SEM) Measurement Procedure

Starch analysis was performed using a Helios 5 PFIB DualBeam microscope (Thermo Fisher Scientific, Waltham, MA, USA). Prior to imaging, the samples were coated with 5 nm of gold using a CCU-010 HV high vacuum compact coating unit (Safematic, Zizers, Switzerland).

### 3.6. The Process of Release of CHA from Starch Tablets

The vacuum-dried starch mass containing AE was milled to prepare tablets with a diameter of 5.0 mm and a weight of 65 ± 5 mg using a Tablet Press TDP 0 (LFA, UK, Bicester). The release profile of chlorogenic acid (CHA) from these tablets was then assessed. Fifty-mL Erlenmeyer flasks were placed on a laboratory shaker set to 50 revolutions per minute. The tablets were transferred into the flasks containing 40 mL of PBS buffer (pH 6.8). The release test was conducted at a temperature of 23 ± 1 °C. Solution samples (3 mL) were collected at specified intervals. The collected solution was filtered using a 0.22 μm Nylon syringe filter and subjected to spectrophotometric analysis at 324 nm (UV-Vis HP 8453 spectrophotometer, Agilent (HP), Santa Clara, CA, USA). Each measurement was performed in triplicate. The collected volumes were returned to the system. The release kinetics were subsequently evaluated using zero-order, first-order, second-order, Higuchi, Korsmeyer–Peppas, and Weibull models.

## 4. Conclusions

Studies conducted on modified starches have demonstrated considerable variability in both structure and function. Each successive modification of native starch affected its properties. The physicochemical performance of starch was not intrinsic to the native polymer but emerged as a direct, quantifiable function of the processing history and modification chemistry. Amine modifications (cationization) provided predictable structural stiffening correlated with substitution levels (Group I), while carboxylate modifications facilitated complex cross-linking that drastically altered colloidal behavior and enhanced stability through multi-site coordination (Group II). Cationization was found to be more effective when a carboxyl group was introduced. The electrophilic carbon atom in the carbonyl group interacted strongly with amino groups, forming robust hydrogen bonds, as evidenced by FTIR, DSC, and TG analyses. Furthermore, annealing increased the reactivity of the carbonyl groups. Annealed cationized citrate starches also exhibited the highest amorphousness, viscosity, thermal stability and resistance to amylolytic enzymes. The observed enhanced material properties required a synergistic interplay between structural disruption (annealing), chemical functionalization (-COOH or -NH_2_), and intermolecular forces (hydrogen bonding/electrostatic attraction). Simple modification seemed to be insufficient; the combination of treatments dictates the final material architecture. When reacting with chlorogenic acid, the most modified starches displayed a delayed yet highly turbulent kinetic profile. This was likely attributed to both the high reactivity of the particles and the subsequent formation of agglomerates; efforts should focus on resolving this issue in future work. The degree of structural disorder, quantified by changes in crystallinity (XRD) and thermodynamic parameters (DSC), served as a robust predictor for functional performance (e.g., increased amorphous content correlated with enhanced API binding and modified gelatinization enthalpy). Processing control over starch structure, e.g., nanostructuring, is thus the paramount determinant when tailoring materials for advanced transfer applications. 

An intriguing alternative is presented by annealed cationic starches, which exhibited significantly lower viscosity than citrate starches and did not form agglomerates. Furthermore, they displayed a promising controlled-release kinetic profile (particularly starch A10). Despite their slightly lower thermal resistance compared to the other starches, they demonstrated an interesting capsule-like structure with pores, suggesting potential for practical applications.

## Figures and Tables

**Table 1:** The physicochemical parameters of the starches from Group I, where G—group, SG—subgroup, NST—nitrogen substitution, ARE—amination reaction efficiency, D[4,3]—hydrodynamic diameter, ER—enzymatic resistance.

G	SG	Starch	NST [%]	ARE [%]	D[4,3] [μm]	pH		Viscosity [mPas]		ER [%]
						25 °C	37 °C	25 °C	37 °C	
I	A	NS0	-	-	41.01	7.439 ± 0.015	7.244 ± 0.008	2.1521 ± 0.0591	1.8251 ± 0.0341	0.11 ± 0.01
		NS5	0.0082 ± 0.0006	16.91	41.01	7.146 ± 0.023	7.271 ± 0.009	2.4181 ± 0.0281	1.6921 ± 0.0741	0.94 ± 0.04
		NS10	0.0111 ± 0.0009	23.05	42.02	6.940 ± 0.016	7.050 ± 0.038	2.6941 ± 0.0111	2.4601 ± 0.0501	2.15 ± 0.02
		NS24	0.0187 ± 0.0004	38.74	49.00	6.931 ± 0.038	6.853 ± 0.119	3.3621 ± 0.0281	2.5331 ± 0.0261	2.19 ± 0.03
	B	A0	-	-	41.07	7.830 ± 0.050	7.859 ± 0.009	2.3961 ± 0.0291	1.9781 ± 0.0261	3.11 ± 0.03
		A5	0.0084 ± 0.0017	17.50	41.90	7.448 ± 0.024	7.268 ± 0.002	2.4292 ± 0.0076	2.0741 ± 0.0101	4.10 ± 0.06
		A10	0.0137 ± 0.0007	28.32	42.66	7.024 ± 0.002	7.003 ± 0.011	2.9251 ± 0.0201	2.3941 ± 0.0101	5.32 ± 0.03
		A24	0.0221 ± 0.0001	45.84	46.16	7.134 ± 0.007	6.897 ± 0.020	3.1406 ± 0.0915	2.9021 ± 0.0281	4.90 ± 0.03

Note. NS0—native starch, NS5, NS10, NS24—native starch cationized for 5, 10 and 24 h, respectively; A0—annealed starch, A5, A10, A24—annealed starch cationized for 5, 10 and 24 h, respectively.

**Table 2:** The physicochemical parameters of the starches from Group II, where G—group, SG—subgroup, NST—nitrogen substitution, ARE—amination reaction efficiency, D[4,3]—hydrodynamic diameter, ER—enzymatic resistance.

G	SG	Starch	NST [%]	ARE [%]	D[4,3] [μm]	pH		Viscosity [mPas]		ER [%]
						25 °C	37 °C	25 °C	37 °C	
II	A	C0	-	-	77.40	4.707 ± 0.001	4.679 ± 0.003	7.5821 ± 0.4791	10.2971 ± 0.1241	12.35 ± 0.05
		C5	0.0309 ± 0.0046	76.73	82.90	5.716 ± 0.004	5.678 ± 0.006	10.1659 ± 1.0143	6.7961 ± 0.1435	13.21 ± 0.03
		C10	0.0415 ± 0.0069	86.09	83.30	7.177 ± 0.002	7.158 ± 0.003	12.3699 ± 0.8220	8.4211 ± 0.2990	22.50 ± 0.13
		C24	0.0268 ± 0.0035	55.59	82.20	6.262 ± 0.008	6.249 ± 0.003	8.4122 ± 0.2605	7.5793 ± 1.0962	17.63 ± 0.03
	B	CA0	-	-	85.30	4.471 ± 0.003	4.456 ± 0.002	19.7531 ± 0.6081	20.4911 ± 2.7471	14.18 ± 0.03
		CA5	0.0313 ± 0.0006	64.93	82.40	5.491 ± 0.010	5.611 ± 0.005	33.5241 ± 1.3511	56.7311 ± 0.8881	17.34 ± 0.03
		CA10	0.0223 ± 0.0006	46.27	84.80	6.129 ± 0.002	6.026 ± 0.011	15.2111 ± 0.2721	15.9821 ± 0.2391	25.90 ± 0.13
		CA24	0.0256 ± 0.0006	53.05	82.40	5.906 ± 0.069	5.747 ± 0.016	13.7361 ± 0.7671	17.4471 ± 0.2021	21.42 ± 0.03

Note. C0—citrate starch, C5, C10, C24—citrate starch cationized for 5, 10 and 24 h, respectively; CA0—annealed citrate starch, CA5, CA10, CA24—annealed citrate starch cationized for 5, 10 and 24 h, respectively.

**Table 3:** The parameters of the diffraction and thermal study of the starch; Cryst—relative crystallinity, Moist—Moisture content, ∆T—the difference between Tg and Tc, ∆H—enthalpy.

Starch	NS0	NS5	NS10	NS24	A0	A5	A10	A24	C0	C5	C10	C24	CA0	CA5	CA10	CA24
Cryst [%]	22.6	26.3	24.9	24.0	26.0	22.8	20.8	23.0	18.9	25.3	20.4	21.7	18.0	17.2	16.8	18.2
Moist [%]	10.7	9.4	8.7	8.8	8.5	8.6	9.3	8.7	5.7	7.7	6.0	7.3	5.3	6.3	5.5	6.2
ΔT [°C]	214	228	225	222	213	219	216	212	229	224	223	224	223	226	222	220
T_g_/T_m_	0.33	0.30	0.32	0.33	0.31	0.31	0.33	0.32	0.23	0.28	0.29	0.28	0.27	0.26	0.28	0.30
ΔH [J/g]	256.2	335.9	330.2	284.6	257.2	232.7	333.9	339.1	209.8	347.6	264.9	348.1	216.7	217.5	263.4	233.8

**Table 4:** Starch types ordered to the groups according to modification process.

Starch Groups		Annealing	Cationization	Esterification/ Cross-Linking
IA	Native Starch (NS0)	NO	NO	NO
	NS0 Cationized for 5 h (NS5)	NO	YES	NO
	NS0 cationized for 10 h (NS10)	NO	YES	NO
	NS0 cationized for 24 h (NS24)	NO	YES	NO
IB	Annealed starch (A0)	YES	NO	NO
	A0 cationized for 5 h (A5)	YES	YES	NO
	A0 cationized for 10 h (A10)	YES	YES	NO
	A0 cationized for 24 h (A24)	YES	YES	NO
IIA	Citrate starch (C0)	NO	NO	YES
	C0 Cationized for 5 h (C5)	NO	YES	YES
	C0 cationized for 10 h (C10)	NO	YES	YES
	C0 cationized for 24 h (C24)	NO	YES	YES
IIB	Annealed C0 (CA0)	YES	NO	YES
	CA0 cationized for 5 h (CA5)	YES	YES	YES
	CA0 cationized for 10 h (CA10)	YES	YES	YES
	CA0 cationized for 24 h (CA24)	YES	YES	YES

## Data Availability

The raw data supporting the conclusions of this article will be made available by the authors on request.

## Appendix A

Kinetic models of chlorogenic acid release from the starch tablets.

**Table A1:** The parameters of kinetic models of chlorogenic acid release from studied starches. Meaning of symbols: K_(0)_, K_(I)_, K_(II)_, K_(H)_, K_(KP)_—rate constants, *n*—exponent of release, β—shape constant, α—scale constant, r^2^—determination coefficient, P—parameters, S—starch, BF—best fit.

	Mathematical Kinetic Models																
	Zero-Order		1st-Order		2nd-Order		Higuchi		Korsmeyer-Peppas				Weibull				BF
P	K_(0)_	SD	K_(I)_	SD	K_(II)_	SD	K_(H)_	SD	K_(KP)_	SD	*n*	SD	β	SD	α	SD	
	[%·min^−1^]		[min^−1^]		[min^−1^%^−1^]		[min^−1/2^%]		[min^-*n*^]		[-]		[-]		[-]		
S	NS0																
	0.3592	0.0665	−0.0153	0.0009	0.0013	0.0009	6.5097	1.0249	0.1067	0.0354	0.5080	0.0879	0.7883	0.1103	0.0564	0.0193	W
r^2^	0.7364		0.7365		0.1620		0.8429		0.8203				0.8730				
S	NS5																
	0.3834	0.0396	−0.0114	0.0041	−0.0008	0.0020	6.7084	0.8014	0.1045	0.1069	0.6609	0.4278	0.8491	0.2676	0.0395	0.0308	H,W
r^2^	0.8557		0.7607		0.3876		0.9097		0.8894				0.9034				
S	NS10																
	0.3388	0.0356	−0.0141	0.0142	−0.0013	0.0016	5.9890	0.5695	0.1819	0.1032	0.3875	0.1641	0.6630	0.0744	0.0749	0.0124	W
r^2^	0.7731		0.6285		0.2857		0.8346		0.7267				0.8388				
S	NS24																
	0.3016	0.1322	−0.0085	0.0052	0.0004	0.0004	5.3273	2.2764	0.1626	0.0972	0.4151	0.1218	0.6166	0.2474	0.0810	0.0612	KP
r^2^	0.7998		0.8491		0.5574		0.8748		0.9689				0.8898				
S	A0																
	0.3761	0.0383	−0.0109	0.0031	0.0007	0.0005	6.6374	0.6887	0.0682	0.0625	0.7573	0.4386	0.8966	0.2904	0.0299	0.0221	W
r^2^	0.8199		0.7563		0.5456		0.8884		0.8589				0.8810				
S	A5																
	0.3505	0.0287	−0.0207	0.0090	−0.0002	0.0031	6.3347	0.5393	0.0828	0.0735	0.6927	0.2363	0.7619	0.1998	0.0715	0.0495	W
r^2^	0.7473		0.8834		0.1235		0.8496		0.8832				0.9334				
S	A10																
	0.2356	0.0651	−0.0027	0.0057	0.0001	0.0001	4.0152	1.1938	0.3007	0.1144	0.2472	0.0970	0.4390	0.1184	0.1067	0.0286	I
r^2^	0.8232		0.8353		0.7518		0.8272		0.6907				0.2121				
S	A24																
	0.3041	0.0728	−0.0069	0.0019	0.0004	0.0005	5.3316	1.1826	0.2265	0.0413	0.2867	0.0463	0.5957	0.1265	0.0791	0.0244	KP
r^2^	0.8329		0.8580		0.5981		0.8928		0.9928				0.9413				
S	C0																
	0.1026	0.0205	−0.0080	0.0098	−0.0014	0.0025	2.0174	0.1147	0.3061	0.2146	0.3681	0.1694	0.2329	0.0767	0.5314	0.4004	KP
r^2^	0.1812		0.4037		0.1745		0.2253		0.5889				0.2255				
S	C5																
	0.1324	0.0246	−0.0131	0.0133	−0.0001	0.0005	2.3974	0.5505	0.4634	0.1216	0.1846	0.0518	0.2644	0.0866	0.3938	0.2213	I
r^2^	0.3125		0.5845		0.1261		0.3508		0.4339				0.3675				
S	C10																
	0.1813	0.0532	−0.0178	0.0108	−0.0021	0.0039	3.2128	0.8187	0.3255	0.1692	0.2824	0.1331	0.3350	0.0664	0.2994	0.1548	I
r^2^	0.4719		0.7595		0.2634		0.5153		0.5289				0.5377				
S	C24																
	0.1181	0.0663	−0.0234	0.0253	0.0001	0.0002	2.2528	1.0804	0.4412	0.2390	0.2123	0.1296	0.2784	0.1180	0.4293	0.3384	I
r^2^	0.3709		0.7218		0.2992		0.4363		0.5942				0.4881				
S	CA0																
	0.2099	0.0516	−0.0136	0.0050	−0.0082	0.0143	3.5707	0.9276	0.4121	0.3267	0.2413	0.1623	0.3228	0.1286	0.3856	0.3406	I
r^2^	0.6074		0.6830		0.3699		0.6132		0.5664				0.6083				
S	CA5																
	0.1515	0.0578	−0.0127	0.0078	0.0006	0.0008	2.7120	0.8515	0.4245	0.2033	0.1972	0.0589	0.3183	0.0796	0.3024	0.1200	I
r^2^	0.3876		0.7260		0.2052		0.4185		0.4907				0.4330				
S	CA10																
	0.1038	0.0215	−0.0111	0.0071	0.0001	0.0001	1.8512	0.2352	0.6029	0.1871	0.1113	0.0463	0.2033	0.0064	0.4490	0.1489	I
r^2^	0.5059		0.7764		0.3900		0.5401		0.5305				0.5546				
S	CA24																
	0.0703	0.0064	−0.0088	0.0063	0.0001	0.0001	1.2319	0.1864	0.6744	0.2716	0.0892	0.0973	0.1338	0.0411	0.5939	0.2223	I
r^2^	0.3540		0.5891		0.3604		0.3414		0.2856				0.3138				
